# Pressuromodulation at the cell membrane as the basis for small molecule hormone and peptide regulation of cellular and nuclear function

**DOI:** 10.1186/s12967-015-0707-6

**Published:** 2015-11-26

**Authors:** Hemant Sarin

**Affiliations:** Freelance Investigator in Translational Science and Medicine, Charleston, WV USA

**Keywords:** Direct CM receptor-mediated stabilizing shift pressuromodulation, Direct CM receptor-mediated stabilizing shift pressuromodulation cum external cationomodulation (≥3+ → 1+), Indirect 1ary CM-mediated shift pressuromodulation (Perturbomodulation), Indirect 2ary CM receptor-mediated shift pressuromodulation (SS 1+), Indirect 3ary CM receptor-mediated shift pressuromodulation (Receptor endocytic 2+), Indirect (pseudo) 3ary CM receptor-mediated shift pressuromodulation (Receptor endocytic hydroxylocarbonyloetheroylomodulation 0)

## Abstract

Building on recent knowledge that the specificity of the biological interactions of small molecule hydrophiles and lipophiles across microvascular and epithelial barriers, and with cells, can be predicted on the basis of their conserved biophysical properties, and the knowledge that biological peptides are cell membrane impermeant, it has been further discussed herein that cellular, and thus, nuclear function, are primarily regulated by small molecule hormone and peptide/factor interactions at the cell membrane (CM) receptors. The means of regulating cellular, and thus, nuclear function, are the various forms of CM Pressuromodulation that exist, which include Direct CM Receptor-Mediated Stabilizing Pressuromodulation, sub-classified as Direct CM Receptor-Mediated Stabilizing Shift Pressuromodulation (Single, Dual or Tri) or Direct CM Receptor-Mediated Stabilizing Shift Pressuromodulation (Single, Dual or Tri) cum External Cationomodulation (≥3+ → 1+); which are with respect to acute CM receptor-stabilizing effects of small biomolecule hormones, growth factors or cytokines, and also include Indirect CM- or CM Receptor-Mediated Pressuromodulation, sub-classified as Indirect 1ary CM-Mediated Shift Pressuromodulation (Perturbomodulation), Indirect 2ary CM Receptor-Mediated Shift Pressuromodulation (Tri or Quad Receptor Internal Pseudo-Cationomodulation: SS 1+), Indirect 3ary CM Receptor-Mediated Shift Pressuromodulation (Single or Dual Receptor Endocytic External Cationomodulation: 2+) or Indirect (Pseudo) 3ary CM Receptor-Mediated Shift Pressuromodulation (Receptor Endocytic Hydroxylocarbonyloetheroylomodulation: 0), which are with respect to sub-acute CM receptor-stabilizing effects of small biomolecules, growth factors or cytokines. As a generalization, all forms of CM pressuromodulation decrease CM and nuclear membrane (NM) compliance (whole cell compliance), due to pressuromodulation of the intracellular microtubule network and increases the exocytosis of pre-synthesized vesicular endogolgi peptides and small molecules as well as nuclear-to-rough endoplasmic reticulum membrane proteins to the CM, with the potential to simultaneously increase the NM-associated chromatin DNA transcription of higher molecular weight protein forms, secretory and CM-destined, mitochondrial and nuclear, including the highest molecular weight nuclear proteins, Ki67 (359 kDa) and Separase (230 kDa), with the latter leading to mitogenesis and cell division; while, in the case of growth factors or cytokines with external cationomodulation capability, CM Receptor External Cationomodulation of CM receptors (≥3+ → 1+) results in cationic extracellular interaction (≥3+) with extracellular matrix heparan sulfates (≥3+ → 1+) concomitant with lamellopodesis and cell migration. It can be surmised that the modulation of cellular, and nuclear, function is mostly a reactive process, governed, primarily, by small molecule hormone and peptide interactions at the cell membrane, with CM receptors and the CM itself. These insights taken together, provide valuable translationally applicable knowledge.

**Electronic supplementary material:**

The online version of this article (doi:10.1186/s12967-015-0707-6) contains supplementary material, which is available to authorized users.

## Background

The specificity of the biological interactions of small molecule hydrophiles and lipophiles can be predicted on the basis of their conserved biophysical properties, which are relative hydrophilicity or lipophilicity in context of molecular size and the distribution of charge over molecular space [1]. Based on these observations, it can be stated that small biomolecules with either cationic or anionic charge are relatively or absolutely restricted to permeation across capillary barriers with restrictive inter-cellular junctional pore complexes [1], as well as excluded from permeation across cell membrane (CM) protein channel aqueous pores in their charged forms in the absence of voltage gating required to increase the channel’s physiologic upper limit of pore size [2, 3], with excess divalent cation, Ca 2+, auto-vesicularizng into the endogolgi smooth endoplasmic reticulum (SER) [4, 5]. In the case of anionic small biomolecule hydrophiles with polyvalent or divalent anionicity such as glutamate, these are impermeant to CM protein channel pores in their polyvalent or divalent anionic forms, closely associate with excess divalent cation, Ca2+, and in association with, also auto-endocytose vesiculize into cell membranes [6], as do cationic small biomolecules such as epinephrine and dopamine due to sufficient poly-univalent cationicity in molecular space (2+ cationicity equivalent: 1+ insufficient separation (IS) 1+), while small biomolecules with charge only traverse CM protein channel pores (i.e. Ca2+ channel pores) across looser channel aqueous pores during voltage gating or oxidative stress, as molecular size permits their passage in these specific instances, which is the case of excess Lactate (1−) generated during metabolic acidosis.

In the case of the endogenous steriods, these include the corticosteroids and sex steroids, the corticosteroids being small molecules of lipophilic character with van der Waals diameters (vdWDs) of ~0.87 nm (nm) and the sex steroid hormones being small molecules of lipophilic character with vdWDs of ~0.80 nm, which permeate across inter-epithelial junction pore complexes via diffusion, while both are restricted to permeation across tight junction vascular/microvascular capillary endothelium [1, 7]. At the individual cell level, the small molecule hormone steroids by being molecular size restricted at CM aqueous pores do not permeate across CM pores, and also do not associate to any significant degree with CM phospholipids, since corticosteroids (cortisol, aldosterone) are asymmetrically polyhydroxylated/carbonylated hormones and the sex steroids (estradiol, progesterone, testosterone, androstenedione) are symmetrically di-hydroxylated/di-carbonylated hormones, with molecular structures favorable to association with CM alpha helix protein receptors in the context of sufficient incorporating lipophilicity for size to do so [8]. As a result of remaining at the cell membrane-to-aqueous interface, the steroids exert their molecular effects at the alpha helix-based CM receptors, whereby, the endogenous corticosteroids primarily exert their effects at the juxtaposed classical GR (97 kDa) and MR (110 kDa) (MR-classical GR complex) [9], by binding to the Mineralocorticoid Receptor (MR) portion of the classical glucocorticoid cum mineralocorticoid receptor complex for which portion they have greater binding affinities [9, 10], while the endogenous sex steroids bind to the Estrogen Receptor (ER) (65 kDa), progestorone receptor (PR) and androgen receptor (AR), in order of greatest to least potency steroid receptor respectively (MR-classical GR > ER > PR > AR) [11, 12]. Moreover, the molecular arrangement of hydrophilic groups attached directly to the immediate steroid backbone is insufficient to induce endocytosis of the respective hormone-receptor complexes, therefore, both classes of steroids are CM receptor pressuromodulators for the specific cell types expressing their receptors *vis a vis* ligand-bound receptor pressuromodulation of the specific receptor’s microtubular network (MR-classical GR, ER, PR or AR)-linked to the receptor’s nuclear chromatin DNA (MR-classical GR, ER, PR or AR) at the level of the nuclear membrane (NM) [13]: The intracellular microtubular network is immobile [14], as opposed to the intracellular F-Actin network, which mobilizes [14], in response to CM receptor-mediated pressuromodulation.

As the chromatin DNA is located along the NM [13], CM pressuromodulation-mediated pressure loading of the specific receptor’s microtubular network-linked to the nuclear membrane (NM)-associated histone-wound DNA chromatin, temporarily unwinds the histone-wound DNA chromatin for transcription, that which upregulates the specific receptor’s expression on the NM-to-RER-to-CM receptor, and importantly, also decreases whole cell cum nuclear compliance, that which results in the immediate exocytosis of, other peptides, both pre-synthesized vesicular Golgi peptide and small molecule forms as well as CM-destined nuclear-to-RER receptor proteins, and concomitantly, simultaneously increases the likelihood of the transcription of higher molecular weight protein forms, secretory and CM receptor, mitochondrial and nuclear, including the highest molecular weight mitogenesis cell division-associated nuclear proteins, Ki67 (359 kDa) and separase (230 kDa): Thus, CM pressuromodulation of whole cell compliance is analogous to mechanical pressure-mediated decreases in whole cell compliance to the biological upper limit of increased intracellular tension [15, 16], for which there must be an upper limit of decreased whole cell compliance required to induce mitogenesis and cell division, and in corollary, that which must be equivalent for all cells, whereby, less compliant cells reach the upper limit faster, while more compliant cells reach the upper limit slower, and, in context of local microenvironment stiffness [17, 18].

As a generalization, the overall character of cell response to direct CM receptor-mediated pressuromodulation is dependent on receptor binding potential (BP), a product of the receptor density (*B*max) and 1/*K*d, and in the case of endogenous small molecule hormones, only in the case of corticosteriod, aldosterone, does the half life at the receptor (t_1/2_ @ receptor) begin to be a significant determinant of pressuromodulation effect, which binds to the MR portion of the MR-classical GR with a sub-nM *K*d, in the decimolar (dM) range, whereby, aldosterone’s t_1/2_ @ receptor is 140 min, which makes it a significant CM pressuromodulator [4, 19–27], despite the fact that only ~170 receptors (MR-Classical GR) are expressed for it on most cell membranes [28–30]; whereas, in the case of the peptides, the half life at the receptor (t_1/2_ @ receptor) stands be a significant determinant of pressuromodulation effect for the monomeric, dimeric or trimeric peptides that bind to polymeric receptors/receptor subunits (IGF-I/II for example [31–35]), those which bind with sub-nM *K*d affinities irrespective of the receptor density.

Based this recent knowledge on the conserved determinants of biological function of small biomolecules in the physiologic state, it can be stated that the biological determinants underlying peptide and factor interactions are just as conserved at the individual cell CM level, which, in turn, regulate cellular, and thus, the nuclear function of a cell. In the commentary, this unifying hypothesis is further explored for the spectrum of small molecule hormones and peptide hormones, including immunomodulatory peptide factors, supported by the findings of published studies, on the structures and functions, of the same.

## Commentary

### Small molecule regulation of intracellular function vis a vis interaction at the cell membrane (CM) receptor

The small biomolecules (<0.5 to 1 nm) include the: (1) small molecule hydrophiles (neutral hydrophiles, neutral cationoneutral hydrophiles, cationic-anionic hydrophiles, anionic cationoneutral hydrophiles, cationic hydrophiles); (2) small molecule hydro-lipophiles (simple cationic hydro-lipophiles, circumferentially polyhydroxylated/carbonylated hydro-lipophile [non-compact (>CM pore size)], circumferentially polyhydroxylated/carbonylated hydro-lipophile + exterior cationicity [non-compact (>CM pore size)]); and (3) small molecule lipophiles (small lipophiles, asymmetric unihydroxylated lipophiles (stable), asymmetric unihydroxylated lipophiles (unstable), asymmetric polyhydroxylated lipophiles (unstable), asymmetric unicarboxylated lipophiles (stable), asymmetric unicarboxylated lipophiles (stable), asymmetric unicarboxylated lipophiles (unstable), asymmetric polyhydroxylated sterols, symmetric dihydroxylated or trihydroxylated/dicarbonylated sterols, symmetric dihydroxylated lipophiles, polyhydroxylated/carbonylated lipophiles (compact), circumferentially polyhydroxylated/carbonylated lipophiles [non-compact (>CM pore size)], circumferentially polyhydroxylated/carbonylated/etheroylated lipophiles [non-compact (>CM pore size)] (Table 1; Additional file 1: Table S1; Additional file 2: Figure S2; Additional file 3: Figure S3; Additional file 4: Figure S4; Additional file 5: Complete Table 1 in Supplemental File Format).

**Table 1:** Conservation of small biomolecule, peptide or factor and non-small molecule non-peptide function

Type	Sub-type	Example(s)	Cell membrane (CM) or CM receptor	Effect at cell membrane (CM) receptor	Intracellular result of effect
Small molecule					
Small molecule hydrophile	Neutral hydrophile	H2O, nitrogenous bases	CM receptor aqueous pore	n/a (subcellular interaction, including nuclear/mitochondrial)	CM receptor/nuclear/mitoch aqueous pore permeation → DNA/RNA
	Neutral cationoneutral hydrophile	Apolar amino acids (i.e. valine, alanine, leucine, isoleucine, etc)	CM receptor aqueous pore	n/a (subcellular interaction, including nuclear/mitochondrial)	CM receptor/nuclear/mitoch aqueous pore permeation → RNA/proteins
	Cationic–anionic hydrophile	Neurotransmitter (glycine, GABA) (IS 1+ 1−)	CM receptor aqueous pore	Receptor aqueous pore non-cationomodulation (isomodulation) and CM non-depolarization	CM receptor aqueous pore permeation
	Anionic cationoneutral hydrophile	Neurotransmitter glutamate (IS 1− 1−)	Peri-CM/peri-CM receptor aqueous pore	Ca2+ pseudo-association	Vesicular non-auto-endocytosis
	Cationic hydrophile	Neurotransmitters norepinephrine (1+), acetylcholine (1+)	CM receptor aqueous pore	Receptor aqueous pore internal cationomodulation and CM depolarization	Depolarization
	Cationic hydrophile	Neurotransmitters norepinephrine (1+), acetylcholine (1+)	CM	Direct CM cationiomodulation (Poly IS 1+)	Vesicular endocytosis
	Cationic hydrophile	Histamine @ acidic pH (IS 1+ 1+)	CM	Direct CM cationiomodulation (2+)	Vesicular auto-endocytosis
Small molecule hydro-lipophile	Simple cationic hydro-lipophile	Neurotransmitters dopamine (1+), seratonin (1+) and histamine (1+)	CM receptor protein	Receptor external cationiomodulation and CM non-depolarization	Non-depolarization
	Simple cationic hydro-lipophile	Neurotransmitters dopamine (1+), seratonin (1+) and histamine (1+)	CM	Direct CM cationiomodulation (poly IS 1+)	Vesicular endocytosis
	Circumferentially polyhydroxylated/carbonylated hydro-lipophile [non-compact (>pore size)]	i.e. Ouabain	CM receptor alpha helix isophilic aqueous pore (i.e. Na/K ATPase)	Receptor external hydroxymodulation → (Pseudo) 3ary indirect shift pressuromodulation	CM interaction receptor endocytosis [mitogenesis]
	Circumferentially polyhydroxylated/carbonylated hydro-lipophile + exterior cationicity [non-compact (>pore size)]	i.e. Doxorubicin (1+)	CM receptor alpha helix isophilic aqueous pore (i.e. Na/K ATPase)	Receptor external hydroxymodulation	CM interaction receptor endocytosis and cationicity (1+)-mediated mitochondrial toxicity
Small molecule lipophile	Small lipophile	i.e. Benzene, di.e.thyl ether	CM receptor aqueous pore	Mitochondrial membrane perturbomodulation → 1ary indirect shift pressuromodulation	Chromatin DNA protein synthesis/exocytosis
	Asymmetric unihydroxylated lipophile (stable)	Cholesterol (3-hydroxycholesterol), i.e. Cholecalciferol (3-hydroxyvitamin D3)	CM	CM incorporopressuromodulation	Baseline Chromatin DNA Protein Synthesis/Exocytosis
	Asymmetric unihydroxylated lipophile (unstable)	i.e. Hexan-1-ol, retinol	CM	CM perturbomodulation → 1ary indirect shift pressuromodulation	Chromatin DNA protein synthesis/exocytosis
	Asymmetric polyhydroxylated lipophile (unstable)	i.e. Phorbol ester 12-O-Tetradecanoylphorbol-13-acetate (TPA)	CM	CM perturbomodulation → 1ary indirect shift pressuromodulation	Chromatin DNA Protein Synthesis/exocytosis
	Asymmetric unicarboxylated lipophile (stable)	Saturated fatty acid → ester	CM	CM incorporopressuromodulation	Baseline chromatin DNA protein synthesis/exocytosis
	Asymmetric unicarboxylated lipophile (stable)	Polyunsaturated fatty acid → ester (i.e. omega-3/6)	CM	CM Incorporo-negativopressuromodulation	n/a
	Asymmetric unicarboxylated lipophile (unstable)	Non-fatty acid (i.e. retinoic acid)	CM	CM perturbomodulation → 1ary indirect shift pressuromodulation	Chromatin DNA protein synthesis/exocytosis
	Asymmetric polyhydroxylated sterol	Aldosterone, dexamethasone, cortisol	CM receptor protein	Receptor stabilizing shift pressuromodulation	Chromatin DNA protein synthesis/exocytosis
	Symmetric di or trihydroxylated/dicarbonylated sterol	Testosterone, estrogen, progesterone	CM receptor protein	Receptor stabilizing shift pressuromodulation	Chromatin DNA protein synthesis/exocytosis
	Symmetric dihydroxylated lipophile	i.e. Calcifediol (1,25-dihydroxyvitamin D3)	CM	CM perturbomodulation → 1ary indirect shift pressuromodulation	Chromatin DNA protein synthesis/exocytosis
	PolyHydroxylated/carbonylated lipophile (compact)	i.e. 3-Isobutyl-1-methylxanthine (IBMX)	CM and CM receptor aqueous pore	CM perturbomodulation → 1ary indirect shift pressuromodulation	Chromatin DNA protein synthesis/exocytosis
	Circumferentially polyhydroxylated/carbonylated lipophile [non-compact (>pore size)]	i.e. Forskolin	CM receptor alpha helix isophilic aqueous pore	Receptor external hydroxymodulation → (pseudo) 3ary indirect shift pressuromodulation	CM interaction receptor endocytosis [mitogenesis]
	Circumferentially polyhydroxylated/carbonylated/etheroylated lipophile [non-compact (>pore size)]	i.e. Paclitaxel (taxol), colchicine	CM receptor alpha helices	Receptor external hydroxymodulation	CM interaction receptor endocytosis and microtubular network disruption
Non-small molecule non-peptide	Polyphospholipid polysaccharide	Lipopolysaccharide (LPS)	CM	CM perturbomodulation → 1ary indirect shift pressuromodulation	Chromatin DNA protein synthesis/exocytosis
Peptide	Small (non-alpha non-beta helix) peptide	Atrial natriuretic peptide (anp) monomer (1+ IS 1+)	CM receptor protein monomer	Receptor external cationiomodulation (2+) → 3ary indirect shift pressuromodulation	Cationic (2+) CM interaction receptor endocytosis [mitogenesis]
	Small (non-alpha non-beta helix) peptide	Bradykinin monomer (1–9 AAs) (1+ IS 1+ SS 1+)	CM receptor protein monomer	Receptor external cationiomodulation (2+) → 3ary indirect shift pressuromodulation	Cationic (2+) CM interaction receptor endocytosis [mitogenesis]
	Small (non-alpha non-beta helix) peptide	Des-Arg9 Bradykinin Monomer (1-8 AAs) (1+ IS 1+)	CM Receptor Protein Monomer	Receptor External Cationiomodulation (2+) → 3ary Indirect Shift Pressuromodulation	Cationic (2+) CM Interaction Receptor Endocytosis [Mitogenesis]
	Small (non-alpha non-beta helix) peptide	Sulfate neutralized lys-bradykinin (kallidin) monomer (1–10 AAs) (IS 3+ → 1+)	CM receptor protein monomer	Receptor stabilizing shift pressuromodulation	Chromatin DNA protein synthesis/exocytosis
	Small (non-alpha non-beta helix) peptide	Vasopressin arginine (anti-diuretic hormone: ADH) monomer (1+)	CM receptor protein monomer	Receptor stabilizing shift pressuromodulation	Chromatin DNA protein synthesis/exocytosis
	Small (non-alpha non-beta helix) peptide	Neurotensin monomer (1+)	CM receptor protein monomer	Receptor stabilizing shift pressuromodulation	Chromatin DNA protein synthesis/exocytosis
	Small (non-alpha non-beta helix) peptide	Angiotensin II monomer	CM receptor protein monomer	Receptor stabilizing shift pressuromodulation	Chromatin DNA protein synthesis/exocytosis
	Small (non-alpha non-beta helix) peptide	Ps4 thyrotropin releasing hormone (TRH) monomer	CM receptor protein monomer	Receptor stabilizing shift pressuromodulation	Chromatin DNA protein synthesis/exocytosis
	Small (non-alpha non-beta helix) peptide	Somatostatin (growth hormone release inhibiting peptide: ghrip) monomer	CM receptor protein monomer	Receptor stabilizing shift pressuromodulation	Chromatin DNA protein synthesis/exocytosis
	Small (non-alpha non-beta helix) peptide	Oxytocin monomer	CM receptor protein monomer	Receptor stabilizing shift pressuromodulation	Chromatin DNA protein synthesis/exocytosis
	Short monoalpha helix	Glucagon monomer	CM receptor protein monomer	Receptor stabilizing shift pressuromodulation	Chromatin DNA protein synthesis/exocytosis
	Short monoalpha helix-loop-short 2-way beta helix	Adrenocorticotropic hormone (ACTH) monomer	CM receptor protein monomer → dimer	Receptor stabilizing shift pressuromodulation	Chromatin DNA protein synthesis/exocytosis
	Long monoalpha helix	Parathyroid hormone (PTH)/PTH releasing peptide (PTHrP) monomer	CM receptor protein monomer	Receptor stabilizing shift pressuromodulation	Chromatin DNA protein synthesis/exocytosis
	Long monoalpha helix-loop-short 2-way beta helix	Pro-ACTH Pro-opiomelanocortin (POMC) monomer	CM receptor protein monomer → dimer	Receptor stabilizing shift pressuromodulation	Chromatin DNA protein synthesis/exocytosis
	Short monoalpha helix-loop-short monoalpha helix	Adrenocorticotrophin releasing hormone/factor (CRH/F) monomer	CM receptor protein dimer (2 su: 3 su)	Dual receptor stabilizing shift pressuromodulation	Chromatin DNA protein synthesis/exocytosis
	Short monoalpha helix-loop-short monoalpha helix	Insulin monomer	CM receptor protein dimer (2 su: 3 su)	Dual receptor stabilizing shift pressuromodulation	Chromatin DNA protein synthesis/exocytosis
	Short monoalpha helix-loop-short monoalpha helix-loop-short monoalpha helix	Osteocalcin monomer	CM receptor protein dimer (2 su: 3 su)	Dual receptor stabilizing shift pressuromodulation	Chromatin DNA protein synthesis/exocytosis
	Short monoalpha helix-loop-short monoalpha helix-loop-short monoalpha helix	Insulin-like growth factor-1 (IGF1/II; somatomedin C) monomer	CM receptor protein dimer (2 su: 3 su)	Dual receptor stabilizing shift pressuromodulation	Chromatin DNA protein synthesis/exocytosis
	Short monoalpha helix-loop-short monoalpha helix-loop-short monoalpha helix	Prolactin (PRL) releasing hormone/factor (PRLRH/factor) monomer	CM receptor protein dimer (2 su: 3 su)	Dual receptor stabilizing shift pressuromodulation	Chromatin DNA protein synthesis/exocytosis
	Short monoalpha helix-loop-short monoalpha helix-loop-short monoalpha helix	Growth hormone releasing hormone/factor (GHRH/F) monomer	CM receptor protein dimer (2 su: 3 su)	Dual receptor stabilizing shift pressuromodulation	Chromatin DNA protein synthesis/exocytosis
	Short monoalpha helix-loop-short monoalpha helix-loop-short monoalpha helix	Gonadotropin releasing hormone/factor (GnRH/F) monomer	CM receptor protein dimer (2 su: 3 su)	Dual receptor stabilizing shift pressuromodulation	Chromatin DNA protein synthesis/exocytosis
	Aligned multialpha helix	Interleukin-3 (IL-3) monomer	CM receptor protein dimer (2 su: 2 su)	Dual receptor stabilizing shift pressuromodulation	Chromatin DNA protein synthesis/exocytosis
	Aligned multialpha helix	Interleukin-7 (IL-7) monomer	CM Receptor Protein Dimer (2 su: 2 su)	Dual receptor stabilizing shift pressuromodulation	Chromatin DNA protein synthesis/exocytosis
	Aligned multialpha helix	Interleukin-12 (IL-12) monomer	CM receptor protein dimer (2 su: 2 su)	Dual receptor stabilizing shift pressuromodulation	Chromatin DNA protein synthesis/exocytosis
	Aligned multialpha helix	Interleukin-23 (IL-23) monomer	CM receptor protein dimer (2 su: 2 su)	Dual receptor stabilizing shift pressuromodulation	Chromatin DNA protein synthesis/exocytosis
	Aligned multialpha helix	Prolactin (PRL) monomer	CM receptor protein dimer (2 su: 2 su)	Dual receptor stabilizing shift pressuromodulation	Chromatin DNA protein synthesis/exocytosis
	Aligned multialpha helix	Growth hormone (GH) monomer	CM receptor protein dimer (2 su: 2 su)	Dual receptor stabilizing shift pressuromodulation	Chromatin DNA protein synthesis/exocytosis
	Aligned multialpha helix	Erythropoietin (Ep) monomer	CM receptor protein dimer (2 su: 2 su)	Dual receptor stabilizing shift pressuromodulation	Chromatin DNA protein synthesis/exocytosis
	Aligned multialpha helix	Interferon gamma (INF-g) homodimer	CM receptor protein trimer (3 su: 3 su: <3 su>)	Dual-to-tri receptor stabilizing shift pressuromodulation	Chromatin DNA protein synthesis/exocytosis
	Semi-aligned multialpha helix cum short monoalpha helix	Interleukin-2 (IL-2) monomer	CM receptor protein dimer (3 su: 2 su)	Dual receptor stabilizing shift pressuromodulation	Chromatin DNA protein synthesis/exocytosis
	Semi-aligned multialpha helix cum short monoalpha helix	Interleukin-6 (IL-6) monomer	CM receptor protein dimer (3 su: 2 su)	Dual receptor stabilizing shift pressuromodulation	Chromatin DNA protein synthesis/exocytosis
	Semi-aligned multialpha helix cum short monobeta 2-way helix	Interleukin-4 (IL-4) monomer	CM receptor protein dimer (4 su: 2 su)	Dual receptor stabilizing shift pressuromodulation	Chromatin DNA protein synthesis/exocytosis
	Semi-aligned multialpha helix cum short monobeta 2-way helix	Interleukin-13 (IL-13) monomer	CM receptor protein dimer (4 su: 2 su)	Dual receptor stabilizing shift pressuromodulation	Chromatin DNA protein synthesis/exocytosis
	Semi-aligned multialpha helix cum short monobeta 2-way helix	Interleukin-5 (IL-5) monomer	CM receptor protein dimer (4 su: 2 su)	Dual receptor stabilizing shift pressuromodulation	Chromatin DNA protein synthesis/exocytosis
	Semi-aligned multialpha helix cum short monobeta 2-way helix	Interleukin-15 (IL-15) monomer	CM receptor protein dimer (4 su: 2 su)	Dual receptor stabilizing shift pressuromodulation	Chromatin DNA protein synthesis/exocytosis
	Semi-aligned multialpha helix cum short monobeta 2-way helix	Interleukin-20 (IL-20) monomer	CM receptor protein dimer (4 su: 2 su)	Dual receptor stabilizing shift pressuromodulation	Chromatin DNA protein synthesis/exocytosis
	Semi-aligned multialpha helix cum short monobeta 2-way helix	Granulocyte monocyte-colony stimulating factor (GM-CSF) monomer	CM receptor protein dimer (4 su: 2 su)	Dual receptor stabilizing shift pressuromodulation	Chromatin DNA protein synthesis/exocytosis
	Semi-aligned multialpha helix cum short monoalpha helix cum short loop	Leukemia inhibitory factor (LIF)/oncostatin (OSM) monomer	CM receptor protein dimer (5 su: 3 su)	Dual receptor stabilizing shift pressuromodulation	Chromatin DNA protein synthesis/exocytosis
	Semi-aligned multialpha helix-loop-short monoalpha helix-loop-short monobeta 2-way helix	Granulocyte-colony stimulating factor (G-CSF)/macrophage (mouse)-colony stimulating factor (M-CSF) homodimer	CM receptor protein dimer (5 su: 3 su)	dual receptor stabilizing shift pressuromodulation	Chromatin DNA protein synthesis/exocytosis
	Short monoalpha helix (neutral) cum compact loose non-aligned 3-way beta-X-2-way beta helix (cationic)	Sulfate neutralized interleukin-16 (IL-16) monomer (IS 3+ → 1+)	CM receptor protein (CD4) monomer	External cationiomodulation and receptor stabilizing shift pressuromodulation	Extracellular matrix interaction and chromatin DNA protein synthesis/exocytosis
	Short monoalpha helix (neutral) cum compact loose aligned 3-way beta helix (cationic)	Sulfate neutralized macrophage inflammatory protein-1 beta (MIP-1 beta: CCL20) monomer (IS 3+ → 1+)	CM receptor protein (CCR5) monomer	External cationiomodulation and receptor stabilizing shift pressuromodulation	Extracellular matrix interaction and chromatin DNA protein synthesis/exocytosis
	Short monoalpha helix (neutral) cum compact loose aligned 3-way beta helix (cationic)	Sulfate neutralized CCL3/CCL4/CCL5 (RANTES) monomer (IS 3+ → 1+)	CM receptor protein (CCR5) monomer	External cationiomodulation and receptor stabilizing shift pressuromodulation	Extracellular matrix interaction and chromatin DNA protein synthesis/exocytosis
	Short angled monoalpha helix (neutral) cum compact loose aligned 3-way beta helix (cationic)	Sulfate neutralized SDF-1 (CXCL-12) monomer (IS 3+ → 1+)	CM receptor protein (CXCR4) monomer	External cationiomodulation and receptor stabilizing shift pressuromodulation	Extracellular matrix interaction and chromatin DNA protein synthesis/exocytosis
	Short angled monoalpha helix (neutral) cum compact loose aligned 3.5-way beta helix (cationic)	Sulfate neutralized interleukin-8 (IL-8) monomer (IS 3+ → 1+)	CM receptor protein (CXCR1) monomer	External cationiomodulation and receptor stabilizing shift pressuromodulation	Extracellular matrix interaction and chromatin DNA protein synthesis/exocytosis
	Compact loose non-aligned multibeta helix (cationic) [+/− alpha helix knob (neutral)]	Sulfate neutralized fibroblast growth factor (FGF-19/FGF-2) monomer ×2 (IS 3+ → 1+: IS 3+ → 1+)	CM receptor protein monomer (3 su) → dimer (3 su: 3 su)	External cationiomodulation and dual receptor stabilizing shift pressuromodulation	Extracellular matrix interaction and chromatin DNA protein synthesis/exocytosis
	Compact loose non-aligned multibeta helix (cationic) [+/− alpha helix knob (neutral)]	Sulfate neutralized hepatocyte growth factor alpha (hgf alpha; scatter factor) monomer ×2 (IS 3+ → 1+: IS 3+ → 1+)	CM receptor protein monomer (3 su) → dimer (3 su: 3 su)	External cationiomodulation and dual receptor stabilizing shift pressuromodulation	Extracellular matrix interaction and chromatin DNA protein synthesis/exocytosis
	Compact loose non-aligned multibeta helix (cationic) [+/− alpha helix knob (neutral)]	Sulfate neutralized epidermal growth factor (EGF) monomer ×2 (IS 3+ → 1+: IS 3+ → 1+)	CM receptor protein monomer (3 su) → dimer (3 su: 3 su)	External cationiomodulation and dual receptor stabilizing shift pressuromodulation	Extracellular matrix interaction and chromatin DNA protein synthesis/exocytosis
	Compact loose non-aligned multibeta helix (cationic) [+/− alpha helix knob (neutral)]	Sulfate neutralized interleukin-1 alpha (IL-1 alpha) monomer ×2 (IS 3+ → 1+: is 3+ → 1+)	CM receptor protein monomer (3 su) → dimer (3 su: 3 su)	External cationiomodulation and dual receptor stabilizing shift pressuromodulation	Extracellular matrix interaction and chromatin DNA protein synthesis/exocytosis
	Compact loose non-aligned multibeta helix (cationic) [+/− alpha helix knob (neutral)]	Sulfate neutralized interleukin-1 beta (il-1 beta) monomer ×2 (IS 3+ → 1+: IS 3+ → 1+)	CM receptor protein monomer (3 su) → dimer (3 su: 3 su)	External cationiomodulation and dual receptor stabilizing shift pressuromodulation	Extracellular matrix interaction and chromatin DNA protein synthesis/exocytosis
	Compact tight semi-aligned multibeta helix (cationic)	Tumor necrosis factor alpha (TNF alpha) homotrimer (SS 1+)	CM receptor protein trimer	Tri receptor internal pseudo-cationomodulation → 2ary indirect shift pressuromodulation	Chromatin DNA protein synthesis/exocytosis [mitogenesis]
	Compact tight aligned multibeta helix (cationic)	Adiponectin homotrimer (SS 1+)	CM receptor protein trimer	Tri receptor internal pseudo-cationomodulation → 2ary indirect shift pressuromodulation	Chromatin DNA protein synthesis/exocytosis [mitogenesis]
	Compact tight aligned multibeta helix (cationic)	RANKL homotrimer (SS 1+)	CM receptor protein trimer	Tri receptor internal pseudo-cationomodulation → 2ary indirect shift pressuromodulation	Chromatin DNA protein synthesis/exocytosis [mitogenesis]
	Aligned long multibeta helix (cationic)	Sulfate neutralized thyroid stimulating hormone alpha and beta (TSH alpha and beta) heterodimer (IS 3+ → 1+: IS 3+ → 1+)	CM receptor protein trimer	Tri receptor stabilizing shift pressuromodulation and external cationiomodulation	Chromatin DNA protein synthesis/exocytosis and extracellular matrix interaction
	Aligned long multibeta helix (cationic)	Sulfate neutralized luteinizing hormone alpha and beta (lh alpha and beta) heterodimer (is 3+ → 1+: IS 3+ → 1+)	CM receptor protein trimer	Tri receptor stabilizing shift pressuromodulation and external cationiomodulation	Chromatin DNA protein synthesis/exocytosis and extracellular matrix interaction
	Aligned long multibeta helix (cationic)	Sulfate neutralized follicle stimulating hormone alpha and beta (fsh alpha and beta) heterodimer (IS 3+ → 1+: IS 3+ → 1+)	CM receptor protein trimer	Tri receptor stabilizing shift pressuromodulation and external cationiomodulation	Chromatin DNA protein synthesis/exocytosis and extracellular matrix interaction
	Aligned long multibeta helix (cationic)	Sulfate neutralized human chorionic gonadotropin alpha and beta (HCG alpha and beta) Heterodimer (IS 3+ → 1+: IS 3+ → 1+)	CM receptor protein trimer	Tri receptor stabilizing shift pressuromodulation and external cationiomodulation	Chromatin DNA protein synthesis/exocytosis and extracellular matrix interaction
	Aligned long multibeta helix (cationic)	Sulfate neutralized brain-derived neurotrophic factor (bdnf) homodimer (IS 3+ → 1+: IS 3+ → 1+)	CM receptor protein trimer	Tri receptor stabilizing shift pressuromodulation and external cationiomodulation	Chromatin DNA protein synthesis/exocytosis and extracellular matrix interaction
	Aligned long multibeta helix (cationic)	Sulfate neutralized nerve growth factor beta (NGFb) homodimer (IS 3+ → 1+: IS 3+ → 1+)	CM receptor protein trimer	Tri receptor stabilizing shift pressuromodulation and external cationiomodulation	Chromatin DNA protein synthesis/exocytosis and extracellular matrix interaction
	Aligned long multibeta helix (cationic)	Sulfate neutralized neurotrophins (nts) homodimer (IS 3+ → 1+: IS 3+ → 1+)	CM receptor protein trimer	Tri receptor stabilizing shift pressuromodulation and external cationiomodulation	Chromatin DNA protein synthesis/exocytosis and extracellular matrix interaction
	Aligned long multibeta helix (cationic) cum short monoalpha helix (neutral)	Transforming growth factor beta (TGF beta) homodimer (SS 1+)	CM receptor protein quatramer	Quad receptor internal pseudo-cationomodulation → 2ary indirect shift pressuromodulation	Chromatin DNA protein synthesis/exocytosis [mitogenesis]
	Aligned long multibeta helix (cationic) cum short monoalpha helix (neutral)	Bone morphogenic protein-2/7 (BMP-2/7) homodimer (SS 1+)	CM receptor protein quatramer	Quad receptor internal pseudo-cationomodulation → 2ary indirect shift pressuromodulation	Chromatin DNA protein synthesis/exocytosis [mitogenesis]
	Aligned long multibeta helix (cationic) cum short monoalpha helix (neutral)	Platelet derived growth factor-BB (PDGF-BB) homodimer (SS 1+)	CM receptor protein quatramer	Quad receptor internal pseudo-cationomodulation → 2ary indirect shift pressuromodulation	Chromatin DNA protein synthesis/exocytosis [mitogenesis]
	Aligned long multibeta helix (cationic) cum short angled monoalpha helix (neutral)	Placenta growth factor (PLGF) homodimer (SS 1+)	CM receptor protein quatramer	Quad receptor internal pseudo-cationomodulation → 2ary indirect shift pressuromodulation	Chromatin DNA protein synthesis/exocytosis [mitogenesis]
	Aligned long multibeta helix (cationic) cum short angled monoalpha helix (neutral)	Vascular endothelial growth factor-A (VEGF-A)/vascular permeability factor (VPF) homodimer (1+ IS 1+ SS 1+ IS 1+)	CM receptor protein quatramer	Quad receptor external cationiomodulation (2+) → 3ary indirect shift pressuromodulation	Cationic (2+) CM interaction receptor endocytosis → mitogenesis
	Compact loose non-aligned multibeta helix-intertwined-non-aligned multialpha helix	Diferric transferrin-(Fe3+ → Fe2+)2 monomers ×2 (3+ SS 3+: 3+ SS 3+ → 2+ SS 2+: 2+ SS 2+)	CM receptor protein (TfR) dimer	Fe2+ receptor external cationiomodulation (2+) → 3ary indirect shift pressuromodulation	Cationic diferric iron (Fe2+) CM interaction receptor endocytosis→ mitogenesis
	Long dualalpha helix (neutral) cum compact loose aligned beta helix cum multishort 2-way beta helix (cationic)	Hemochromatosis protein (HPE) monomers ×2 [(1+ IS 1+)n SS (1+ IS 1+)n]	CM receptor protein (TfR) dimer	Transient RSP; external beta helix CM receptor cationiomodulation (2+) → 3ary indirect shift pressuromodulation	Cationic diferric iron (Fe2+) cm interaction receptor endocytosis→ mitogenesis
	Short monoalpha helix-loop-short monoalpha helix-loop-short monoalpha helix (neutral) cum compact loose non-aligned 5-way beta-X-3-way beta helix (cationic)	Partially sulfate neutralized procollagen I peptide III C monomer (IS 4+ → 2+)	CM receptor protein dimer (3 su: 3 su)	Dual receptor external cationiomodulation (2+) → 3ary indirect shift pressuromodulation	Cationic (2+) CM interaction receptor endocytosis → mitogenesis

*SS* sufficient separation of 2+ cationicity in molecular space, which is important, as it precludes Heparan Sulfate neutralization of cationicity, that which requires the presence of >2+ charge insufficiently separated in molecular space

*IS* insufficient separation of 1+ cationicity in molecular space, which is important, as it precludes endocytosis, that which requires the presence of 2+ charge insufficiently separated in molecular space

The specific roles that small molecules play in the biological system is determinable on the basis of an assessment of conserved biophysical properties, relative hydrophilicity or lipophilicity in context of molecular size, as per the predicted octanol-to-water partition coefficient (OWPC)-to-van der Waals diameter (vdWD) ratio (nm^−1^) in context of the predicted vdWD (nm) [1] (Additional file 1: Table S1).

### Neurotransmitter small molecule hydrophile and hydro-lipophile regulation of intracellular function vis a vis interaction at the cell membrane (CM) receptor

The small molecule hydrophiles include the cationic neurotransmitters acetylcholine (1+) and norepinephrine (1+), which are (pure) hydrophiles due to the presence of singular cationicity (1+) cum hydroxylated hydrophilicity in the absence of cell membrane (CM) receptor protein binding lipophilicity. These, being singularly cationic (pure) hydrophiles are internal cationomodulators that cause cell membrane depolarization via the insertion of 1+ cationicity into protein receptor channel aqueous pores (Table 1; Additional file 1: Table S1; Additional file 2: Figure S2; Additional file 5: Complete Table 1 in Supplemental File Format) and potentiate the circumferential propagation of current in effector cell membranes, that which in the case of smooth and cardiac muscle cells results in contraction, and that which in the case of adrenal medulla cells results in depolarization-coupled exocytosis of epinephrine.

The small molecule cationic hydro-lipophile neurotransmitters dopamine (1+) and seratonin (1+), being hydro-lipophiles due to the combinatory presence of singular cationicity (1+), hydroxylated hydrophilicity and receptor protein binding lipophilicity, in contrast to the small molecule cationic hydrophile neurotransmitters (i.e. norepinephrine, acetylcholine), function as mild external cationomodulators, via lipophilic incorporation into CM receptor hydrophobic cores of commensurate lipophilicity on the basis of the incorporating lipophilicity for size of their non-cationic and non-hydroxylated portion, and thus, non-insert positive charge, that which results in non-depolarization of the CM (Table 1; Additional file 1: Table S1; Additional file 3: Figure S3; Additional file 5: Complete Table 1 in Supplemental File Format): Interaction with neuronal CM receptors in such a manner, results in decreased post-synaptic depolarization and contribute significantly towards regulating the tonicity of upper motor neuron-to-lower motor neuron meshwork of inter-neuronal connections [36, 37], and furthermore, in the case of pituitary lactotrophs results in inhibition of prolactin secretion [38], most likely due to competitive antagonism of prolactin releasing hormone (PRL), the shift pressuromodulator of lactotroph CM PRL receptors.

In the case of both the cationic hydrophile internal cationomodulators (acetylcholine and norepinephrine) and the cationic hydro-lipophile mild external cationomodulators (i.e. dopamine and seratonin), both classes with singular cationicity (1+), in the absence of protein channel aqueous pores and receptors to insert into (former) or bind to (latter), such (pure) hydrophiles and hydro-lipophiles, respectively, are reactively endocytosed by pre-synaptic neuronal cell membranes and vesicularize for subsequent re-release due to the concentration of poly 1+ charges per unit volume (Table 1; Additional file 5: Complete Table 1 in Supplemental File Format).

### Non-neurotransmitter hydro-lipophile regulation of intracellular function vis a vis interaction at the cell membrane (CM) receptor

The non-neurotransmitter small molecule hydro-lipophiles include circumferentially polyhydroxylated/carbonylated hydro-lipophiles [non-compact (>pore size)] such as ouabain (Na+/K+ ATPase receptor channel), and circumferentially polyhydroxylated/carbonylated hydro-lipophiles + exterior cationicity [non-compact (>pore size)] such as doxorubicin (Na+/K+ ATPase receptor channel), which are circumferentially hydroxylated and/or carbonylated hydrophiles with core lipophilicity, and therefore, pro-endocytic hydro-lipophiles [39, 40] via receptor hydroxymodulation/carbonylomodulation of receptor channel pores (Table 1; Additional file 1: Table S1; Additional file 3: Figure S3; Additional file 5: Complete Table 1 in Supplemental File Format).

The potential for the non-neurotransmitter small molecule hydro-lipophiles to be pro-endocytic at CM receptor channels is attributable to the presence of incorporating lipophilicity, in the concomitant presence of interacting polyhydroxylated/carbonylated hydrophilicity. As such, both of the circumferentially hydroxylated weak hydrophiles with core lipophilicity, ouabain and doxorubicin, interact with CM protein receptor alpha helix cum alpha helix isophilic aqueous pores, for example, such as those of the Na+/K+ ATPase, they de-stabilize the CM interaction of the multialpha helix constructs of such trans-membrane proteins with internal isophilic aqueous pores, that which results in ligand-bound CM protein endocytosis.

Upon endocytosis, ouabain and doxorubicin, differentially modulate intracellular function: ouabain functions as a indirect (pseudo) 3ary CM receptor-mediated shift pressuromodulator (receptor endocytic hydroxylocarbonyloetheroylomodulation: 0) to decrease whole cell compliance significantly, that which results in the increased exocytosis of the pre-synthesized Golgi peptides as well as RER receptor proteins to the CM, and concomitantly, in the increased protein transcription of additional highest molecular weight (MW) forms (i.e. fibronectin: 240 kDa), including that of the highest molecular weight nuclear proteins, Ki67 (359 kDa) and separase (230 kDa), that which results in mitogenesis, in the concomitant presence of serum [41] [indirect (pseudo) 3ary CM receptor-mediated shift pressuromodulation (receptor endocytic hydroxylocarbonyloetheroylomodulation: 0)] (Additional file 1: Table S1; Additional file 3: Figure S3).

In contrast to ouabain, and importantly in contrast, doxorubicin, with greater interior lipophilicity sufficient to stably associate with the internal little alpha helix of the mitochondrial membrane (MM) voltage-dependent anion channel (VDAC) [42] in context of the concomitant presence of 1+ cationicity, functions more as a chemoxenobiotic hydro-lipophile, by virtue of its ability to anchor mitochondria via non-recruitment of gamma-Tubulin to the MM VDAC [43], that which results in MM disruption and liberation of MM apoptosis inducing factor (AIF), which binds X-linked inhibitor of apoptosis factor (XIAF), freeing XIAF from its association with Caspace-3, the cumulative effect of intracellularly endocytosed doxorubicin, mitochondrial dissolution-mediated cytotoxic cell death [40] (receptor endocytic cationohydroxylocarbonyloetheroylomodulation: 1+) (Additional file 1: Table S1; Additional file 3: Figure S3).

### Polyhydroxylated small molecule sterol regulation of intracellular function vis a vis interaction at the cell membrane (CM) receptor

The polyhydroxylated small molecule lipophile sterols include the: (1) asymmetrically polyhydroxylated sterols, the corticosteroids, aldosterone and cortisol, which are asymmetrically hydroxylated sterols (Table 1; Additional file 1: Table S1; Additional file 4: Figure S4; Additional file 5: Complete Table 1 in Supplemental File Format) [8]; and (2) symmetrically di/trihydroxylated sterols, the sex steroids, testosterone, estrogen and progesterone, which are symmetrically dipolarly di/trihydroxylated sterols (Table 1; Additional file 1: Table S1; Additional file 4: Figure S4; Additional file 5: Complete Table 1 in Supplemental File Format) [8].

The polyhydroxylated small molecule lipophile sterols have the potential to associate with cell membrane protein receptors of commensurate lipophilcity, on the basis of the incorporating lipophilicity for size of the non-hydroxylated portion, which is of lipophilic character, and interacts with the CM receptor protein itself, while the hydroxylated hydrophilicity of the hydroxylated portion, which is of hydrophilic character, interacts with the hydrophilicity of exteriorly hydrophilic microenvironment [10], both of which [8, 40], in concert, are co-determinants of the binding affinity, and importantly, character of the polyhydroxylated small biomolecule interaction with its respective receptor. As such, the asymmetrically hydroxylated lipophiles (corticosteroids) and the symmetrically dipolarly di/trihydroxylated sterols (sex steroids), both being polyhydroxylated small biomolecules with greater incorporating lipophilicity-to-interacting hydroxylated hydrophilicity ratios (corticosteroid and sex steroid hormones) are anti-endocytic for their hormone-receptor complexes and direct CM receptor stabilizing pressuromodulators [8, 11, 20, 22, 23, 44–50] (direct CM receptor-mediated stabilizing shift pressuromodulation: 0) (Table 1; Additional file 1: Table S1; Additional file 3: Figure S3; Additional file 4: Figure S4; Additional file 5: Complete Table 1 in Supplemental File Format).

### Polyhydroxylated/carbonlylated/etheroylated small molecule non-sterol lipophile regulation of intracellular function vis a vis Interaction at the cell membrane (CM) receptor

The polyhydroxylated small molecule non-sterol lipophiles include the: (1) circumferentially polyhydroxylated/carbonylated lipophiles [non-compact (>pore size)] such as forskolin, and (2) circumferentially polyhydroxylated/carbonylated/etheroylated lipophiles [non-compact (>pore size)] such as paclitaxel (taxol) and colchicine, which are circumferentially hydroxylated and/or carbonylated hydrophiles with core lipophilicity, and therefore, pro-endocytic lipophiles (Table 1; Additional file 1: Table S1; Additional file 4: Figure S4; Additional file 5: Complete Table 1 in Supplemental File Format), via CM protein receptor hydroxymodulation/carbonylomodulation/etheroylation.

The potential for polyhydroxylated small molecule non-sterol lipophiles to be pro-endocytic at the CM receptor is attributable to the presence of incorporating lipophilicity in the concomitant presence of interacting polyhydroxylated/carbonylated/etheroylated hydrophilicity, as is the case for the non-neurotransmitter small molecule hydro-lipophiles (Additional file 1: Table S1; Additional file 3: Figure S3). However, the important distinction between the two categories, is that in the case of the polyhydroxylated small molecule non-sterol lipophiles, there is the presence of greater incorporating lipophilicity relative to the interacting hydroxylated hydrophilicity; while the presence of the greater incorporating lipophilicity makes these biomolecules, small molecule Lipophiles, the similar amount of circumferential hydrophilicity enables interaction with CM protein receptor alpha helixes cum alpha helix isophilic aqueous pores (Additional file 1: Table S1; Additional file 4: Figure S4), sufficient enough to de-stabilize the CM interaction of the multialpha helix constructs of such trans-membrane proteins, that which results in ligand-bound CM protein endocytosis.

Upon endocytosis, forskolin and paclitaxel/colchicine, diffferentially modulate intracellular function: Forskolin functions as a indirect (pseudo) 3ary CM receptor-mediated shift pressuromodulator to decrease whole cell compliance significantly, that which results in the increased exocytosis of the pre-synthesized Golgi peptides as well as RER receptor proteins to the CM, and concomitantly, in the increased protein transcription of additional highest molecular weight forms (i.e. fibronectin: 240 kDa) [51], including that of the highest molecular weight nuclear proteins, Ki67 (359 kDa) and Separase (230 kDa), resulting in mitogenesis and cell division, in the concomitant presence of serum [52] [indirect (pseudo) 3ary CM receptor-mediated shift pressuromodulation (receptor endocytic hydroxylocarbonyloetheroylomodulation: 0)] (Additional file 1: Table S1; Additional file 4: Figure S4).

In contrast to forskolin, and importantly in contrast to, both paclitaxel (Taxol) (0) and colchicine (0), function more as cytotoxic lipophiles, due to the presence of greater incorporating lipophilicity than that of Forskolin, in the absence of cationicity, and therefore, the further ability to associate with the intracellular microtubular network forming protein, tubulin beta [53], both paclitaxel and colchicine, are not (pseudo) 3ary CM receptor shift pressuromodulators: instead, by inhibition of tubulin polymerization re-polymerization, both paclitaxel (taxol) and Colchicine exert significant mitochondrial membrane (MM) oxidative stress, via mitochondria anchorage during attempted movement [54, 55], that which results in MM disruption and in cytotoxic cell death via mitochondrial dissolution [40] (receptor endocytic hydroxylocarbonyloetheroylomodulation: 0) (Additional file 1: Table S1; Additional file 4: Figure S4).

### Unihydroxylated and dihydroxylated small molecule lipophile regulation of intracellular function vis a vis interaction directly within the cell membrane (CM)

The asymmetric unihydroxylated small molecule lipophiles include, the cholesterols, 3beta-hydroxycholesterol, and the vitamin D form, cholecalciferol (3-hydroxyvitamin D3), which are unipolarly hydroxylated lipophiles, and directly incorporate into cell membrane (CM) phospholipid layers due to commensurate lipophilcity for size to that of the phospholipid fatty acid-ester moieties, and function as CM stabilizing incorporopressuromodulators that maintain the baseline compliance state of all cell membranes [56] (Table 1; Additional file 1: Table S1; Additional file 4: Figure S4; Additional file 5: Complete Table 1 in Supplemental File Format).

The symmetric di-hydroxylated non-sterols constitute unstable sterol forms such as calcifediol (1, 25-dihydroxyvitamin D3), which neither stably associate with CM proteins or CM bilayers; instead, such unstable small molecule lipophile forms tend to perturb cell membrane lipid layers, and function as CM perturbomodulators, first, by increasing CM compliance slightly, enough to cause the non-exocytosis and intracellular build-up of pre-synthesized Golgi reservoir non-collagenase (MMP)-higher molecular weight protein forms (i.e. fibronectin: 240 kDa; tyrosinase containing-melanosomes) that remain intracellular, secondary to which, whole cell compliance decreases significantly, then resulting in the increased exocytosis of the pre-synthesized Golgi peptides (i.e. fibronectin: 240 kDa) and nuclear-to-RER receptor proteins to the CM, as well as in the increased protein transcription of the spectrum of synthesizable proteins, including in the increased synthesis of the lowest molecular weight protein forms (i.e. osteocalcin: 6 kDa) [57] [1ary indirect shift pressuromodulation (perturbomodulation)] (Table 1; Additional file 1: Table S1; Additional file 4: Figure S4; Additional file 5: Complete Table 1 in Supplemental File Format).

Other notable 1ary indirect CM-mediated shift pressuromodulators are the other unstable phospholipid layer non-incorporating small molecule lipophile forms, the asymmetrically polyhydroxylated forms such as phorbol ester 12-O-tetradecanoylphorbol-13-acetate (TPA) [51] and the similar phorbol esters with fatty acid-ester tails (i.e. phorbol 12,13-dibutyrate) [58], the asymmetrically unihydroxylated forms such as Retinol and asymmetrically unicarboxylated Retinoic Acid [59, 60], the asymmetrically dihydroxylated/carbonylated forms such as blebbistatin [61], as well as the compact polyhydroxylated and/or carbonylated forms such as isobutylmethyl xanthine (IBMX) [62] [1ary indirect shift pressuromodulation (perturbomodulation)] (Table 1; Additional file 1: Table S1; Additional file 4: Figure S4; Additional file 5: Complete Table 1 in Supplemental File Format).

Along these lines, the other types of 1ary indirect shift pressuromodulation (perturbomodulation) include mild primary hypoxia [63, 64] due to primary decreases the rates of mitochondrial oxidative phosphorylation, as well as mild secondary hypoxia due to the secondary decreases the rates of mitochondrial oxidative phosphorylation viz a viz the intracellular effects of small lipophile toxins/toxicants such as benzene and diethyl ether, which due to small molecular sizes also interact non-selectively with the sub-cellular mitochondrial membrane (MM) electron transport chain (ETS) proteins resulting in secondary whole cell ATP deficiency: In both cases of mild hypoxia, primary and secondary, the overall initial effect is an increase in CM compliance due to mild ATP deficiency-mediated decreases in CM protein subunit and CM cohesiveness, that which results in intra-cellular protein build-up towards a decrease in whole cell compliance, and upon acclimatization/recovery, the overall effect being the 1ary indirect CM-mediated shift pressuromodulation effect (perturbomodulation) (Table 1; Additional file 1: Table S1; Additional file 4: Figure S4; Additional file 5: Complete Table 1 in Supplemental File Format).

### Non-small molecule non-peptide regulation of intracellular function vis a vis interaction at the cell membrane (CM) receptor

The non-small molecule non-peptide biomolecules include bacterial cell wall lipopolysaccharide (LPS) comprised of lipid A (phospholipid fatty acid-ester-glycerol moiety equivalent), inner core (phosphorylated poly/monosaccharide phospholipid head phosphotidylcholine/ehtanolamine/serine equivalent) and outer core-to-’O Antigen’ (short-to-long polysaccharide) in series, with the molecular weight of the rough core LPS beginning in the range of 2–5 kDa [65]: LPS is a potent 1ary indirect shift pressuromodulator of the CM, by interdigitating insertion association of its LPS Lipid A component into cell membrane phospholipid layer, which, by first increasing CM compliance slightly, enough to cause the non-exocytosis intracellular build-up of pre-synthesized Golgi reservoir non-collagenase (MMP)-higher molecular weight protein forms (i.e. fibronectin: 240 kDa), and upon a significant decrease in whole cell compliance, secondarily, causes the increased exocytosis of the pre-synthesized Golgi peptides (i.e. fibronectin: 240 kDa) as well as RER receptor proteins to the CM [66], and concomitantly, in the chromatin DNA transcription of the lower and higher molecular weight protein forms, both mitochondrial and nuclear, including the highest molecular weight nuclear proteins, Ki67 (359 kDa) and separase (230 kDa), with the ability to induce mitogenesis and cell division [67, 68] [1ary indirect CM-mediated shift pressuromodulation effect (perturbomodulation)] (Table 1; Additional file 5: Complete Table 1 in Supplemental File Format).

### Small (non-alpha non-beta helix) peptide regulation of intracellular function vis a vis interaction at the cell membrane (CM) receptor

The small (non-alpha non-beta) peptides include the low molecular weight peptides [1–1.6 kDa; 9–14 amino acids (AAs)], without and with tertiary structure intramolecular disulfide bonds, that bind to so-called G protein-coupled receptors (GPRCs), which are multiple alpha helix-based CM receptor constructs that do not dimerize in response to small (non-alpha non-beta) peptide ligand binding, but may co-exist as juxtaposed dimers on the CM [69, 70] (direct CM receptor-mediated stabilizing shift pressuromodulation: 0) (Table 1; Additional file 5: Complete Table 1 in Supplemental File Format).

The small (non-alpha non-beta) di-cationic (1+ IS 1+) peptides include bradykinin (1+ IS 1+ SS 1+) [71] and atrial natriuretic peptide (1+ IS 1+) [72–74] and function as transient pressuromodulators cum external cationomodulators, which incorporate into CM receptor hydrophobic cores of commensurate lipophilcity on the basis of the incorporating lipophilicity for size of their non-cationic portion in the concomitant presence of tertiary structure 1+ cationicity insufficiently separated (IS) in molecular space (1+ IS 1+); thus, via external interaction with the cell membrane phospholipid heads, cause CM and CM receptor endocytosis and vesiculo-vacuolization-through-and-through diaphragmed fenestration of endothelial cells resulting in microvascular capillary hyperpermeability [75], in which case the external cationomodulation-mediated endocytic transformation process decreases endothelial cell compliance significantly, that which, not only results in an almost immediate significant increase in the exocytosis of RER endothelial NOS (eNOS) to the CM and increased nitric oxide (NO) [76], a potent competitive antagonist of smooth muscle cell O_2_ at the electron transport chain (ETS) and in endothelial cell-mediated smooth muscle relaxation and vascular-microvascular vasodilation [71, 72, 75], but also in a significant increase in protein transcription of the entire spectrum of synthesizable proteins, importantly, the highest molecular weight nuclear proteins, Ki67 (359 kDa) and Separase (230 kDa), and in mitogenesis and endothelial cell division [77] [indirect 3ary CM receptor-mediated shift pressuromodulation (single or dual) receptor endocytic external cationomodulation: 2+)] (Table 1; Additional file 5: Complete Table 1 in Supplemental File Format).

The mono-cationic (1+) and non-cationic small (non-alpha non-beta) peptides such as vasopressin arginine (1+) (anti-diuretic hormone), neurotensin (1+) [78], angiotensin II [47, 79], Ps4 thyrotropin releasing hormone [80, 81], somatostatin (growth hormone release inhibiting peptide) and oxytocin incorporate into CM receptor hydrophobic cores of commensurate lipophilcity, thus, via insertion, stabilize receptor G protein-coupled protein receptor monomers and shift pressuromodulate cell membranes by decreasing CM compliance sufficiently enough to cause the increased synthesis and exocytosis of the higher molecular proteins, but not yet the lower molecular weight proteins, and therefore, masquerade as release inhibiting peptides (direct CM receptor-mediated stabilizing shift pressuromodulation: 1+) (Table 1; Additional file 5: Complete Table 1 in Supplemental File Format).

### Monoalpha helix peptide and loop-interconnected dual/poly monoalpha helix peptide regulation of intracellular function vis a vis interaction at the cell membrane (CM) receptor(s)

The monoalpha helix peptides are the singular short and long alpha helix (monoalpha helix) peptides, the singular alpha helix peptides with a short 2-way beta helix tail peptides (monoalpha helix-loop-short 2-way beta helix), and the peptides with loop-interconnected two or more singular alpha helixes (short monoalpha helix-loop-short monoalpha helix, short monoalpha helix-loop-short monoalpha helix-loop-short monoalpha helix), which are of greater molecular weights than the small (non-alpha non-beta) peptides (>1.6 to <14 kDa) without tertiary structure disulfide bonds, which, like the low molecular weight peptides (<1.5 to 2 kDa) (Table 1; Additional file 5: Complete Table 1 in Supplemental File Format).

The short and long monoalpha helix peptides include glucagon and parathyroid hormone (PTH)/PTH releasing peptide (PTHrP) [82–84] that bind to the multiple alpha helix-based Class A GPCRs, which do not dimerize in response to monoalpha helix peptide ligand binding, but provide greater stability to the ligand-receptor complex than the low molecular weight peptides (<1.5 to 2 kDa), and also to bind to alpha helix cum 2-way-X-2-way beta helix-based Class B GPRCs [83], which can dimerize in response to monoalpha helix peptide ligand binding; in comparison the small (non-alpha non-beta) peptides (Neural or 1+), are more effective CM receptor and CM pressuromodulators than the low molecular weight peptides, longer monoalpha helix peptides (PTH/PTHrP) [85, 86] more effective than shorter monoalpha helix peptides (Glucagon) [87] (direct CM receptor-mediated stabilizing shift pressuromodulation) (Table 1; Additional file 5: Complete Table 1 in Supplemental File Format).

The short and long monoalpha helix-loop-short 2-way beta helix peptides such as adrenocorticotropic hormone (ACTH) [88–91] and pro-ACTH pro-opiomelanocortin (POMC) [92], respectively, which, in contrast to the short and long monoalpha helix peptides (without 2-way beta helix tails), bind to one G protein-coupled receptor via the singular alpha helix motif with the potential to dimerize another G protein-coupled receptor via the 2-way beta helix tail, which are known to be expressed on cell membranes in close proximity to one another [70], and thus, provide greater stability to the ligand-receptor complex than the short and long monoalpha helix peptides (without the 2-way beta helix tail) that engage only one G protein-coupled receptor, therefore, in comparison, more effective CM receptor, and CM, pressuromodulators (direct CM receptor-mediated stabilizing shift pressuromodulation) (Table 1; Additional file 5: Complete Table 1 in Supplemental File Format).

The short monoalpha helix-loop-short monoalpha helix peptides include adrenocorticotrophin releasing hormone/factor (CRH/F) [78] and insulin [31, 93–102], and the short monoalpha helix-loop-short monoalpha helix-loop-short monoalpha helix peptides include osteocalcin [103], insulin-like growth factor-1 (IGF1/II; somatomedin C) [31, 34, 35, 93, 104, 105], prolactin (PRL) releasing hormone/factor (PRLRH/factor) [106], growth hormone releasing hormone/factor (GHRH/F) [107] and gonadotropin releasing hormone/factor (GnRH/F), which, in contrast to the short and long monoalpha helix peptides (glucagon, PTH) and the short and long monoalpha helix-loop-short 2-way beta helix monoalpha helix peptides (ACTH, POMC), bind to mixed alpha cum beta helix-construct non-G protein-coupled tyrosine kinase type receptors that homodimerize in response to loop-interconnected dual/poly singular alpha helix peptide binding and adopting a condensed globular conformation, which therefore, function as more effective shift pressuromodulators at the cell membrane than the monoalpha helix peptides (direct CM receptor-mediated stabilizing shift pressuromodulation) (Table 1; Additional file 5: Complete Table 1 in Supplemental File Format).

### Aligned multialpha helix peptide and semi-aligned multialpha helix peptide regulation of intracellular function vis a vis interaction at the cell membrane (CM) receptor(s)

The mulialpha helix peptides are the aligned multiple alpha helix peptides (aligned multialpha helix), the semi-aligned multiple alpha helix peptides (semi-aligned multialpha helix cum short monoalpha helix), the semi-aligned multiple alpha helix with a short singular beta 2-way helix peptides (semi-aligned multialpha helix cum short monobeta 2-way helix), the semi-aligned multiple alpha helix with a short singular alpha helix and short end-loop (semi-aligned multialpha helix cum short monoalpha helix cum short loop), and the peptides with a loop-interconnected semi-aligned multiple alpha helix, short singular alpha helix and short singular beta 2-way helix (semi-aligned multialpha helix-loop-short monoalpha helix-loop-short monobeta 2-way helix) (<30 kDa in monomeric form) (Table 1; Additional file 5: Complete Table 1 in Supplemental File Format).

The aligned multialpha helix peptides include interleukin-3 (IL-3) [108], interleukin-7 (IL-7) [109], interleukin-12 (IL-12) [110, 111], interleukin-23 (IL-23) [112], prolactin (PRL) [113, 114], growth hormone (GH) [115–121] and erythropoietin (Ep) monomers, which function as dual CM receptor stabilizing shift pressuromodulators by stabilizing heteromeric amorphous loop cum beta helix-rich receptor subunits (2 su: 2 su), as well as Interferon gamma (INF-g) homodimer (25 kDa × 2) [122–131], which function as dual-to-tri CM receptor stabilizing shift pressuromodulators by stabilizing heteromeric amorphous loop cum beta helix-rich receptor subunits (3 su: 3 su: <3 su>), and of the aligned multialpha helix peptides, interferon gamma (INF-g) being a homodimer with the potential for receptor trimerization, functions as a more effective pressuromodulator, that which stimulates increased protein transcription of the higher molecular weight proteins including the highest molecular weight, secreted, Fibronectin (240 kDa) [128, 129], as well as nuclear, separase (230 kDa) and Ki67 (359 kDa), thus, with the potential to be mitogenic for certain cell types (with greater baseline compliance) [130, 131] (direct CM receptor-mediated stabilizing shift pressuromodulation) (Table 1; Additional file 5: Complete Table 1 in Supplemental File Format).

The semi-aligned multialpha helix cum short monoalpha helix peptides include interleukin-2 (IL-2) [111, 132, 133] and interleukin-6 (IL-6) [134] monomers, which function as effective dual CM receptor stabilizing shift pressuromodulators by stabilizing heteromeric amorphous loop cum beta helix-rich receptor subunits (3 su: 2 su) (direct CM receptor-mediated stabilizing shift pressuromodulation) (Table 1; Additional file 5: Complete Table 1 in Supplemental File Format).

The semi-aligned multialpha helix cum short monoBeta 2-way helix peptides include interleukin-4 (IL-4) [135, 136], interleukin-13 (IL-13), interleukin-15 (IL-15) [111, 137], interleukin-20 (IL-20) and granulocyte monocyte-colony stimulating factor (GM-CSF) [138–142] monomers, which function as effective dual CM receptor stabilizing shift pressuromodulators by stabilizing heteromeric beta helix-rich receptor subunits (4 su: 2 su) (direct CM receptor-mediated stabilizing shift pressuromodulation) (Table 1; Additional file 5: Complete Table 1 in Supplemental File Format).

The semi-aligned multialpha helix cum short monoalpha helix cum short loop peptides include the leukemia inhibitory factor (LIF)/oncostatin (OSM) [143] monomer, which function as effective dual CM receptor stabilizing shift pressuromodulators by stabilizing heteromeric amorphous loop cum beta helix-rich receptor subunits (5 su: 3 su) (direct CM receptor-mediated stabilizing shift pressuromodulation) (Table 1; Additional file 5: Complete Table 1 in Supplemental File Format).

The semi-aligned multialpha helix-loop-short monoalpha helix-loop-short monobeta 2-way helix peptides include the granulocyte-colony stimulating factor (G-CSF)/macrophage (mouse)-colony stimulating factor (M-CSF) homodimer (60 kDa) [144–147], which functions as effective dual CM receptor stabilizing shift pressuromodulators by stabilizing heteromeric beta helix-rich receptor subunits (3 su: 3 su) (direct CM receptor-mediated stabilizing shift pressuromodulation) (Table 1; Additional file 5: Complete Table 1 in Supplemental File Format).

### Compact loose non-aligned multibeta helix, compact disperse non-aligned multibeta helix peptide and compact tight aligned/non-aligned multibeta helix peptide regulation of intracellular function vis a vis Interaction at the cell membrane (CM) receptor(s)

The compact mulibeta helix peptides are the compact loose non-aligned/aligned multiple beta helix peptides with a short singular non-angled/angled alpha helix (short monoalpha helix cum compact loose non-aligned 3-way beta-X-2-way beta helix, short monoalpha helix cum compact loose aligned 3-way beta helix, short angled monoalpha helix cum compact loose aligned 3-way beta helix, short angled monoalpha helix cum compact loose aligned 3.5-way beta helix), the compact loose non-aligned multiple beta helix peptides ± an ancillary alpha knob (compact loose non-aligned multibeta helix ± alpha helix knob), and the compact tight semi-aligned/aligned multiple beta helix peptides (compact tight semi-aligned multibeta helix, compact tight aligned multibeta helix) (<35 kDa in monomeric form) (Table 1; Additional file 5: Complete Table 1 in Supplemental File Format).

The compact loose non-aligned multibeta helix cum short monoalpha helix peptides include sulfate neutralized CD4 receptor ligand interleukin-16 (IL-16) (IS 3+ → 1+) [148–151], sulfate neutralized CCR5 receptor ligands macrophage inflammatory protein-1 beta (MIP-1 beta: CCL20) (IS 3+ → 1+), CCL3, CCL4 and CCL5 (RANTES) (IS 3+ → 1+) [152, 153], sulfate neutralized CXCR4 receptor ligand SDF-1 (CXCL-12) (IS 3+ → 1+) [152] and sulfate neutralized CXCR1 ligand interleukin-8 (IL-8) (IS 3+ → 1+) [154, 155], all monomeric, which, function as CM receptor stabilizing shift pressuromodulators by binding to alpha helix-rich chemokine receptor cores via neutral short alpha helix or short angled alpha helix association for the pressuromodulating effect, and exceptionally effective external cationomodulators by draping over receptor exteriors via cationic compact loose non-aligned multibeta helix motifs (3-way beta-X-2-way beta helix, 3-way beta helix or 3.5-way beta helix) for the external cationomodulation effect (3+ → 1+) [direct CM receptor-mediated stabilizing shift pressuromodulation (single, dual or tri) cum external cationomodulation (≥3+ → 1+)], that which results in exocytosis and transcription of higher molecular weight protein forms, and CM-to-extracellular matrix interactions associated with lamellopodesis and cell migration, respectively (Table 1; Additional file 5: Complete Table 1 in Supplemental File Format).

The compact loose non-aligned Multibeta helix ± alpha helix knob peptides include sulfate neutralized fibroblast growth factor (FGF-19/FGF-2) (IS 3+ → 1+) [102, 156–162], sulfate neutralized hepatocyte growth factor alpha (HGF alpha; Scatter Factor) (IS 3+ → 1+) [163, 164], sulfate neutralized epidermal growth factor (EGF) (IS 3+ → 1+) [165, 166], sulfate neutralized Interleukin-1 alpha (IL-1 alpha) (IS 3+ → 1+) [167] and sulfate neutralized interleukin-1 beta (IL-1 beta) (IS 3+ → 1+) [156, 168], each monomers, however, capable of binding as separate monomers (IS 3+ → 1+: IS 3+ → 1+) to opposite sides of beta-rich receptors close in molecular space, which dimerize (3 su: 3 su), and thus, which function as effective CM receptor stabilizing shift pressuromodulators [direct CM receptor-Mediated stabilizing shift pressuromodulation (single, dual or tri) cum external cationomodulation (≥3+ → 1+)], that which results in exocytosis and transcription of higher molecular weight protein forms, and exceptionally effective external cationomodulators via cationic extracellular interaction (≥3+) with extracellular matrix heparan sulfate neutralizaiton of excess cationicity (≥3+ → 1+), that which results in lamellopodesis and in cell migration (Table 1; Additional file 5: Complete Table 1 in Supplemental File Format).

The compact tight semi-aligned/aligned Multibeta helix peptides include tumor necrosis factor alpha (TNF alpha) (SS 1+) [164, 169–173], adiponectin (SS 1+) [174] and RANKL (SS 1+) [175–180] with the ability to self-trimerize without superimposition, and concomitantly, trimerize beta helix-based receptors, which, therefore, function as exceptionally effective pressuromodulators, indirect shift pressuromodulators, as a result of prolonged pseudo-cationic (1+) association with the CM secondary to self-trimerization and receptor trimerization, that which increases CM compliance sufficiently enough to cause the non-exocytosis of pre-synthesized Golgi reservoir collagenase (MMP)-insensitive higher molecular weight protein forms (i.e. Fibronectin: 240 kDa), which remain intracellular, the process of which results in a secondary significant decrease in whole cell compliance [indirect 2ary CM receptor-mediated shift pressuromodulation (tri or quad receptor internal pseudo-cationomodulation: SS 1+)], that which results in significantly increased protein transcription of the entire spectrum of synthesizable proteins, importantly, the highest molecular weight nuclear proteins, Ki67 (359 kDa) and separase (230 kDa), and in mitogenesis and cell division (Table 1; Additional file 5: Complete Table 1 in Supplemental File Format).

### Aligned long multibeta helix peptide regulation of intracellular function vis a vis interaction at the cell membrane (CM) receptor(s)

The aligned long mulibeta helix peptides are the aligned long multiple beta helix peptides (aligned long multibeta helix), the aligned long multiple beta helix peptides with a short singular alpha helix (aligned long multibeta helix cum short monoalpha helix), and the aligned long multiple beta helix with a short angled singular alpha helix (aligned long nultibeta helix cum short angled monoalpha helix) (<25 kDa in monomeric form) (Table 1; Additional file 5: Complete Table 1 in Supplemental File Format).

The aligned long multibeta helix peptides include the heterodimeric sulfate neutralized glycosylated hormones, thyroid stimulating hormone alpha and beta (TSH alpha and beta) (IS 3+ → 1+: IS 3+ → 1+), luteinizing hormone alpha and beta (LH alpha and beta) (IS 3+ → 1+: IS 3+ → 1+), follicle stimulating hormone alpha and beta (FSH alpha and beta) (IS 3+ → 1+) [181–183], human chorionic gonadotropin alpha and beta (HCG alpha and beta) (IS 3+ → 1+: IS 3+ → 1+) [184–187], brain-derived neurotrophic factor (BDNF) (IS 3+ → 1+: IS 3+ → 1+) [188] and nerve growth factor beta (NGFb) (IS 3+ → 1+: IS 3+ → 1+) at the p75 NGF receptor (higher *B*max) [189–192], which, by binding to amorphous loop cum beta-rich receptor subunits and receptor trimerization, function as effective CM receptor stabilizing shift pressuromodulators to induce the Golgi vesicular exocytosis of various small molecule hormones (i.e. thyroxine, testosterone, estrogen and progesterone, etc.) that require significant pressuromodulation to exocytose [8], and concomitantly, function as external cationomodulators due to the presence of solvent accessible cationic amino acid R groups that interact with extracellular matrix heparan sulfates (IS 3+ → 1+: IS 3+ → 1+) that which results in lamellopodesis and in cell migration [193, 194] [direct CM receptor-mediated stabilizing shift pressuromodulation (single, dual or tri) cum external cationomodulation (≥3+ → 1+)] (Table 1; Additional file 5: Complete Table 1 in Supplemental File Format).

The aligned long multibeta helix cum short/short angled monoalpha helix peptides include the non-superimposed interlocking homodimeric peptides, transforming growth factor beta (TGF beta) (SS 1+) [195–209], bone morphogenic protein-2/7 (BMP-2/7) (SS 1+) [199, 210], platelet derived growth factor-BB (PDGF-BB) (SS 1+) [131, 211–213] and placenta growth factor (PLGF) (SS 1+) [214], with solvent accessible cationic amino acid R groups sufficiently separated (SS) in molecular space (1+/R group), which, by interlocking to each other via the neutral short monoalpha helix motif, and by concomitantly interacting with beta helix-based receptor via the cationic aligned long multibeta helix motif (SS 1+), function as exceptionally effective pressuromodulators, indirect shift pressuromodulators, as a result of prolonged pseudo-cationic (1+) association with the CM secondary to beta helix-based receptor quatramerization, that which increases CM compliance sufficiently enough to cause the non-exocytosis of pre-synthesized Golgi reservoir collagenase (MMP)-insensitive higher molecular weight protein forms (i.e. Fibronectin: 240 kDa), which remain intracellular, the process of which results in a secondary significant decrease in whole cell compliance [indirect 2ary CM receptor-mediated shift pressuromodulation (tri or quad receptor internal pseudo-cationomodulation: SS 1+)], that which results in significantly increased protein transcription of the entire spectrum of synthesizable proteins, importantly, the highest molecular weight nuclear proteins, Ki67 (359 kDa) and Separase (230 kDa), and in mitogenesis and cell division (Table 1; Additional file 5: Complete Table 1 in Supplemental File Format).

The aligned long MultiBeta helix cum short angled monoalpha helix peptides also include the non-superimposed interlocking homodimeric peptide, vascular endothelial growth factor-A (VEGF-A)/vascular permeability factor (VPF) (1+ IS 1+ SS 1+ IS 1+) [162, 215–219], which, in contrast to TGF beta, BMP-2/7 and PDGF-BB, possesses solvent accessible cationic amino acid R groups with mono-cationicity insufficiently separated (IS) in molecular space (1+/R group IS 1+/R group), thus, similarly to the small (non-alpha non-beta) di-cationic peptides such as the bradykinin monomer (1 + IS 1 +) [indirect 3ary CM receptor-mediated shift pressuromodulation (single or dual receptor endocytic external cationomodulation: 2+)], functions as a transient pressuromodulator cum external cationomodulator, but in contrast to bradykinin, is significantly more effective as an external cationomodulator (2+), as a result of prolonged cationic association with the CM by binding to its beta helix-based receptor, the KDR receptor, as an interlocked homodimer in context of KDR receptor quatramerization, that which results in external cationomodulation (2+)-mediated endocytic endothelial cell transformation with a concomitant significant decrease in endothelial cell compliance [indirect 3ary CM receptor-mediated shift pressuromodulation (single or dual receptor endocytic external cationomodulation: 2+)] and increase in protein transcription of the entire spectrum of synthesizable proteins, importantly, the highest molecular weight nuclear proteins, Ki67 (359 kDa) and Separase (230 kDa), and in mitogenesis and endothelial cell division (Table 1; Additional file 5: Complete Table 1 in Supplemental File Format).

### Mixed helix and combinatory helix peptide regulation of intracellular function vis a vis interaction at the cell membrane (CM) receptor(s)

The mixed helix peptides include the transferrin receptor (TfR) [220] peptides, transferrin-(Fe3+ → Fe2+)_2_ [221], a compact loose non-aligned multibeta helix (neutral)-intertwined-non-aligned multialpha helix peptide (Fe3+ SS Fe3+ → Fe2+ SS Fe2+), and hemochromatosis protein (HPE) [221], a long dualalpha helix (neutral) cum compact loose aligned beta helix cum multishort 2-way beta helix peptide [(1+ IS 1+)n SS (1+ IS 1+)n], and the combinatory helix peptides include the liver sinusoidal endothelial cell (LSEC) mannose receptor peptide, partially sulfate neutralized procollagen I peptide III C monomer (IS 4+ → 2+) [13, 222–225], a short monoalpha helix-loop-short monoalpha helix-loop-short monoalpha helix (neutral) cum compact loose non-aligned 5-way beta-X-3-way beta helix (cationic) (Table 1; Additional file 5: Complete Table 1 in Supplemental File Format).

The mixed helix transferrin receptor peptides, transferrin-(Fe3+ → Fe2+)_2_ and Hemochromatosis Protein, as monomers, are capable of binding as separate monomers to opposite sides of juxtaposed mixed alpha-rich cum beta-rich receptors via neutral alpha helix ligand-to-alpha helix receptor association, which, due to the presence of di-cationicity, the acquired Fe2+ in the former (transferrin), and the inherent 2+ in the later (HPE) (MultiShort 2-way beta helix), function as effective external cationomodulators (2+) via external cationomodulation (2+)-mediated endocytosis, the process of which results in significant endocytosis-vesiculovacuolization-endothelial cell fenestration, particularly, at the liver sinusoidal endothelial cell (LSEC) where the concentration of hepatocyte-produced Transferrin and HPE is the greatest, and thus, maintains the highly endocytic-endothelial glycocalyx layer-devoid reticuloendothelial LSEC phenotype [7, 221], the concomitant presence of VEGF only required to maintain the openly fenestrated LSEC phenotype (versus diaphragm fenestrated) [7, 226]; furthermore, the secondary significant decrease in endothelial cell compliance from transferrin and HPE external cationomodulation (2+)-mediated endocytosis [indirect 3ary CM receptor-mediated shift pressuromodulation (single or dual receptor endocytic external cationomodulation: 2+)], results in the increased protein transcription, importantly, that of the highest molecular weight nuclear proteins, Ki67 (359 kDa) and separase (230 kDa), that which results in mitogenesis and cell division [4], and in LSEC turnover, in the physiologic state (Table 1; Additional file 5: Complete Table 1 in Supplemental File Format).

The combinatory helix peptides, partially sulfate neutralized procollagen I peptide III C monomer (IS 4+ → 2+), consists of: (1) a compact loose non-aligned 5-way beta-X-3-way beta helix (cationic) motif, and in comparison to interleukin-16 (IL-16), due to the presence of 5-way beta (versus an IL-16 3-way beta helix) criss-crossed by 3-way beta helix (versus an IL-16 2-way beta helix), has 4+ cationicity insufficiently separated in molecular space (IS) (versus an IL-16 3+), in which case 3+ cationicity of the 4+ is effectively neutralized by heparan sulfate (or hyaluronate/glucoronate) in systemic circulation (IS 4+ → 1+), but at the reticuloendothelial liver sinusoidal endothelial cell (LSEC), which do not have a thick endothelial glycocalyx layer (- heparan sulfate/hyaluronate/glucoronate), the excess cationicity is not effectively neutralized (IS 4+ → 2+), that which, specifically, results in endocytosis, and uptake, of partially sulfate neutralized procollagen I peptide III C monomer (IS 4+ → 2+) at the LSEC ‘cation-dependent’ and cation-independent ‘Mannose’ receptors [224, 225] (Table 1); and (2) a short monoalpha helix-loop-short monoalpha helix-loop-short monoalpha helix (Neutral) motif, and in comparison to insulin-like growth factor (IGF) for example [31, 32], very similar, whereby, the procollagen I peptide III C peptide, binds with ‘Mannose’ receptors with decimolar (dM) affinity (*K*d) [33] viz a viz tight association with the neutral alpha helix amorphous loop cum beta helix complex of the receptor construct [227], also very similar to the insulin-like growth factor receptor (IGFR) for example [33, 34]; furthermore, the secondary significant decrease in LSEC compliance from partially sulfate neutralized procollagen I peptide III C monomer (IS 4+ → 2+) external cationomodulation (2+)-mediated endocytosis [indirect 3ary CM receptor-mediated shift pressuromodulation (single or dual receptor endocytic external cationomodulation: 2+)], results in the increased protein transcription of the highest molecular weight nuclear proteins, Ki67 (359 kDa) and Separase (230 kDa), and at the LSEC, importantly, that which results in LSEC mitogenesis and cell division, and in turnover, in the physiologic state, while, when present in the fully neutralized (IS 4+ → 1+) form in vitro (heparin-containing media) functions, instead, as a direct CM receptor-mediated stabilizing shift pressuromodulator cum external cationomodulator (≥3+ → 1+) [227, 228] (Table 1; Additional file 5: Complete Table 1 in Supplemental File Format).

## Conclusion

Building on recent knowledge that the specificity of the biological interactions of small molecule hydrophiles and lipophiles across microvascular and epithelial barriers, and with cells, can be predicted on the basis of their conserved biophysical properties, and the knowledge that biological peptides are cell membrane impermeant, it has been further discussed herein that cellular, and thus, nuclear function, are primarily regulated by small molecule hormone and peptide/factor interactions at the cell membrane (CM) receptors.

The means of regulating cellular, and thus, nuclear function, are the various forms of CM Pressuromodulation that exist, which include direct CM receptor-mediated stabilizing pressuromodulation, sub-classified as direct CM receptor-mediated stabilizing shift pressuromodulation (single, dual or tri) or direct CM receptor-mediated stabilizing shift pressuromodulation (single, dual or tri) cum external cationomodulation (≥3+ → 1+); which are with respect to acute CM receptor-stabilizing effects of small biomolecule hormones, growth factors or cytokines, and also include indirect CM- or CM receptor-mediated pressuromodulation, sub-classified as indirect 1ary CM-mediated shift pressuromodulation (perturbomodulation), indirect 2ary CM receptor-mediated shift pressuromodulation (tri or quad receptor internal pseudo-cationomodulation: SS 1+), indirect 3ary CM receptor-mediated shift pressuromodulation (single or dual receptor endocytic external cationomodulation: 2+) or indirect (Pseudo) 3ary CM receptor-mediated shift pressuromodulation (receptor endocytic hydroxylocarbonyloetheroylomodulation: 0), which are with respect to sub-acute CM receptor-stabilizing effects of small biomolecules, growth factors or cytokines.

As a generalization, all forms of CM pressuromodulation decrease CM and nuclear membrane (NM) compliance (whole cell compliance), due to pressuromodulation of the intracellular microtubule network and increases the exocytosis of pre-synthesized vesicular endogolgi peptides and small molecules as well as nuclear-to-rough endoplasmic reticulum membrane proteins to the CM, with the potential to simultaneously increase the NM-associated chromatin DNA transcription of higher molecular weight protein forms, secretory and CM-destined, mitochondrial and nuclear, including the highest molecular weight nuclear proteins, Ki67 (359 kDa) and separase (230 kDa), with the latter leading to mitogenesis and cell division; while, in the case of growth factors or cytokines with external cationomodulation capability, CM receptor external cationomodulation of CM receptors (≥3+ → 1+) results in cationic extracellular interaction (≥3+) with extracellular matrix heparan sulfates (≥3+ → 1+) concomitant with lamellopodesis and cell migration.

It can be surmised that the modulation of cellular, and nuclear, function is mostly a reactive process, governed, primarily, by small molecule hormone and peptide interactions at the cell membrane, with CM receptors and the CM itself. These insights taken together, provide valuable translationally applicable knowledge.

## Additional files

10.1186/s12967-015-0707-6 Conserved biophysical properties of small molecule hydrophiles, hydro-lipophiles and lipophiles.
10.1186/s12967-015-0707-6 Small molecule hydrophiles.
10.1186/s12967-015-0707-6 Small molecule hydro-lipophiles.
10.1186/s12967-015-0707-6 Small molecule lipophiles.
10.1186/s12967-015-0707-6 Complete Table 1 in Supplemental File Format.
