# Supplementary material for: Pressuromodulation at the cell membrane as the basis for small molecule hormone and peptide regulation of cellular and nuclear function
Source: J Transl Med. 2015 Nov 26;13:372. doi: 10.1186/s12967-015-0707-6 (PMC4660824; doi:10.1186/s12967-015-0707-6)
Supplement: Supplementary file 1 — 10.1186/s12967-015-0707-6 Conserved biophysical properties of small molecule hydrophiles, hydro-lipophiles and lipophiles. [file 12967_2015_707_MOESM1_ESM.pdf]

Table S1. Conserved Biophysical Properties of Small Molecule Hydrophiles, Hydro-lipophiles and Lipophiles

SMALL MOLECULE HYDROPHILES	Sub-Category & Example(s)	Molecular Formula	Log OWPC	OWPC	Weight (Daltons)	Volume (Ang3)	vdWD (nm)	Psa	Charge Distribution	(Log) OPWC-to-vdWD Ratio (per nm [nm-1])	Cell Membrane (CM) or CM Receptor	Effect at Cell Membrane (CM) Receptor	Intracellular Result of Effect
	Neutral Hydrophile												
	Nitrogenous Base Cytosine	C4H5N3O	-1.4	3.981E-02	111	92	0.55	67	0	-2.5	CM Receptor Aqueous Pore	n/a (Subcellular Interaction, including Nuclear/Mitochondrial)	CM Receptor/Nuclear/Mitoch Aqueous Pore Permeation -> DNA/RNA/Proteins
	Neutral Cationoneutral Hydrophile												
	Amino Acid Valine	C5H11NO2	-1.1	7.943E-02	117	119	0.60	63	0 1+ IS 1-	-1.8	CM Receptor Aqueous Pore	n/a (Subcellular Interaction, including Nuclear/Mitochondrial)	CM Receptor/Nuclear/Mitoch Aqueous Pore Permeation -> RNA
	Cationic-Anionic Hydrophile												
	Neurotransmitter Glycine	C9H13NO3	-2.65	2.239E-03	75	68	0.50	63	1+ IS 1-	-5.3	CM Receptor Aqueous Pore	Receptor Aqueous Pore Non-Cationomodulation (Isomodulation) & CM Non-Depolarization	CM Receptor Aqueous Pore Permeation
	Anionic Cationoneutral Hydrophile												
	Neurotransmitter Glutamate	C5H8NO4	-5.5	3.162E-06	146	127	0.62	108	1+ IS 1- (Ca 2+) 1-	-8.9	Peri-CM/Peri-CM Receptor Aqueous Pore	Ca2+ Pseudo-Association	Vesicular Auto-Endocytosis
	Cationic Hydrophile												
	Neurotransmitter Norepinephrine	C8H11NO3	-2.65	2.239E-03	169	153	0.66	87	1+ (2ary N)	-4.0	CM Receptor Aqueous Pore<br>CM	Receptor Aqueous Pore Internal Cationomodulation & CM Depolarization<br>Direct CM Cationiomodulation (Poly IS 1+)	Depolarization<br>Vesicular Endocytosis
	Neurotransmitter Acetylcholine	C7H16NO2	-3.7	1.995E-04	146	158	0.66	26	1+ (Quat N)	-5.6	CM Receptor Aqueous Pore<br>CM	Receptor Aqueous Pore Internal Cationomodulation & CM Depolarization<br>Direct CM Cationiomodulation (Poly IS 1+)	Depolarization<br>Vesicular Endocytosis

SMALL MOLECULE HYDRO-LIPOPHILES	Sub-Category & Example(s)	Molecular Formula	Log OWPC	OWPC	Weight (Daltons)	Volume (Ang3)	vdWD (nm)	Psa	Charge Distribution	(Log) OPWC-to-vdWD Ratio (per nm [nm-1])	Cell Membrane (CM) or CM Receptor	Effect at Cell Membrane (CM) Receptor	Intracellular Result of Effect
	Simple Cationic Hydro-lipophile												
	Neurotransmitter Dopamine	C8H11NO2	-1.7	1.995E-02	153	145	0.64	66	1+ (2ary N)	-2.6	CM Receptor Protein<br>CM	Receptor External Cationiomodulation & CM Nondepolarization<br>Direct CM Cationiomodulation (Poly IS 1+)	Non-depolarization<br>Vesicular Endocytosis
	Neurotransmitter Seratonin	C10H12N2O	-1.35	4.467E-02	176	163	0.67	62	1+ (2ary N)	-2.0	CM Receptor Protein<br>CM	Receptor External Cationiomodulation & CM Nondepolarization<br>Direct CM Cationiomodulation (Poly IS 1+)	Non-depolarization<br>Vesicular Endocytosis
	Circumferentially PolyHydroxylated/Carbonylated Hydro-lipophile [Non-Compact (>Pore Size)]												
	Ouabain	C29H44O12	-2.78	1.660E-03	585	530	0.99	206	0 (PolyOH cum C=O)	-2.8	CM Receptor Alpha Helix Isophilic Aqueous Pore (ie Na/K ATPase)	Receptor External Hydroxymodulation -> (Pseudo) 3ary Indirect Shift Pressuromodulation	CM Interaction Receptor Endocytosis [Mitogenesis]
	~Ouabain Lipophilic Core	C28H44O3	5.83	6.761E+05	429	441	0.93	~0	0 (Core)	6.3	CM Receptor Alpha Helix	n/a	n/a
	Circumferentially PolyHydroxylated/Carbonylated Hydro-lipophile + Exterior Cationicity [Non-Compact (>Pore Size)]												
	Doxorubicin	C27H29NO11	-0.79	1.622E-01	544	463	0.95	206	0 (PolyOH cum C=O) IS 1+	-0.8	CM Receptor Alpha Helix Isophilic Aqueous Pore (ie Na/K ATPase)	Receptor External Hydroxymodulation	CM Interaction Receptor Endocytosis & Cationicity (1+)-Mediated Mitochondrial Toxicity
	~Doxorubicin Lipophilic Core	C19H18O	4.8	6.310E+04	262	246	0.77	~0	0 (Core)	6.3	CM Receptor Alpha Helix	n/a	n/a

SMALL MOLECULE LIPOPHILES	Sub-Category & Example(s)	Molecular Formula	Log OWPC	OWPC	Weight (Daltons)	Volume (Ang3)	vdWD (nm)	Psa	Charge Distribution	(Log) OPWC-to-vdWD Ratio (per nm [nm-1])	Cell Membrane (CM) or CM Receptor	Effect at Cell Membrane (CM) Receptor	Intracellular Result of Effect
	Small Lipophile												
	Benzene	C6H6	1.97	9.333E+01	78	82	0.53	0	0	3.7	CM & CM Receptor Aqueous Pore	CM Perturbomodulation & 1ary Indirect Shift Pressuromodulation	Chromatin DNA Protein Synthesis/Exocytosis
	Diethyl Ether	C4H10O	0.87	7.413E+00	74	88	0.55	0	0	1.6	CM & CM Receptor Aqueous Pore	CM Perturbomodulation & 1ary Indirect Shift Pressuromodulation	Chromatin DNA Protein Synthesis/Exocytosis
	Asymmetric UniHydroxylated Lipophile (Stable)												
	Cholesterol (3-hydroxycholesterol)	C27H46O	7.11	1.288E+07	387	428	0.92	20	0 (OH)	7.7	CM	CM Incorporopressuromodulation	Baseline Chromatin DNA Protein Synthesis/Exocytosis
	Cholecalciferol (3-hydroxyvitamin D3)	C27H44O	7.13	1.349E+07	384	424	0.92	20	0 (OH)	7.7	CM	CM Incorporopressuromodulation	Baseline Chromatin DNA Protein Synthesis/Exocytosis
	Asymmetric UniHydroxylated Lipophile (Unstable)												
	Hexan-1-ol	C6H14O	1.69	4.898E+01	102	122	0.61	20	0 (OH)	2.8	CM	CM Perturbomodulation & 1ary Indirect Shift Pressuromodulation	Chromatin DNA Protein Synthesis/Exocytosis
	Retinol	C20H30O	4.69	4.898E+04	286	310	0.83	20	0 (OH)	5.7	CM	CM Perturbomodulation & 1ary Indirect Shift Pressuromodulation	Chromatin DNA Protein Synthesis/Exocytosis
	Asymmetric PolyHydroxylated Lipophile (Unstable)												
	Phorbol Ester 12-O-Tetradecanoylphorbol-13-acetate (TPA)	C36H56O8	5.69	4.898E+05	617	614	1.04	130	0 (PolyOH cum 14C Fatty Acid-Ester)	5.5	CM	CM Perturbomodulation & 1ary Indirect Shift Pressuromodulation	Chromatin DNA Protein Synthesis/Exocytosis
	Asymmetric UniCarboxylated Lipophile (Stable)												
	PI Saturated Fatty-Acid Ester Palmitic Fatty Acid (C16)-Ester	C16H30O2	5.79	6.166E+05	254	286	0.81	37	0 (COOEster)	7.2	CM	CM Incorporopressuromodulation	Baseline Chromatin DNA Protein Synthesis/Exocytosis
	PI Polyunsaturated Fatty-Acid Ester g-Linolenic Fatty Acid (18:3 Omega-6)-Ester	C18H30O2	6.06	1.148E+06	278	304	0.82	37	0 (COOEster)	7.4	CM	CM Incorporonegativopressuromodulation	n/a
	Asymmetric UniCarboxylated Lipophile (Unstable)												
	Non-PI Non-Fatty Acid Retinoic Acid	C20H28O2	1.7	5.012E+01	300	312	0.83	37	1- (COO-)	2.0	CM	CM Perturbomodulation & 1ary Indirect Shift Pressuromodulation	Chromatin DNA Protein Synthesis/Exocytosis
	Retinol/Retinoic Acid Lipophilic Core	C20H30	5.97	9.333E+05	270	302	0.82	0	0 (Core)	7.3	CM	CM Perturbomodulation & 1ary Indirect Shift Pressuromodulation	Chromatin DNA Protein Synthesis/Exocytosis
	Asymmetric PolyHydroxylated Sterol												
	Aldosterone	C21H28O5	1.06	1.148E+01	360	341	0.86	92	0 (Asymmetric Poly OH)	1.2	CM Receptor Protein	Receptor Stabilizing Shift Pressuromodulation	Chromatin DNA Protein Synthesis/Exocytosis
	Dexamethasone	C22H29FO	1.68	4.786E+01	393	362	0.87	95	0 (Asymmetric Poly OH)	1.9	CM Receptor Protein	Receptor Stabilizing Shift Pressuromodulation	Chromatin DNA Protein Synthesis/Exocytosis
	Cortisol	C21H30O5	1.28	1.905E+01	362	347	0.86	95	0 (Asymmetric Poly OH)	1.5	CM Receptor Protein	Receptor Stabilizing Shift Pressuromodulation	Chromatin DNA Protein Synthesis/Exocytosis
	Receptor Binding Lipophilic Sterol Backbone Core Range	n/a	5.09-5.85	n/a	244-299	282-327	0.79-0.84	0	Sterol Ring w/ CH3(s)	6.47-6.93	CM Receptor Protein Hydrophobic Core	n/a	n/a
	Symmetric DiHydroxylated/DiCarbonylated Sterol												
	Testosterone	C19H28O2	3.37	2.344E+03	288	294	0.81	37	0 (OH SS OH)	4.1	CM Receptor Protein	Receptor Stabilizing Shift Pressuromodulation	Chromatin DNA Protein Synthesis/Exocytosis
	Estradiol	C18H24O2	3.75	5.623E+03	272	270	0.79	40	0 (OH SS OH)	4.7	CM Receptor Protein	Receptor Stabilizing Shift Pressuromodulation	Chromatin DNA Protein Synthesis/Exocytosis
	Progesterone	C21H30O2	4.15	1.413E+04	314	321	0.84	34	0 (C=O SS C=O)	4.9	CM Receptor Protein	Receptor Stabilizing Shift Pressuromodulation	Chromatin DNA Protein Synthesis/Exocytosis
	Symmetric DiHydroxylated Lipophile												
	Calcifediol (1,25-dihydroxyvitamin D3)	C27H44O2	5.65	4.467E+05	400	432	0.93	41	0 (Symmetric Di-OH)	6.1	CM	CM Perturbomodulation & 1ary Indirect Shift Pressuromodulation	Chromatin DNA Protein Synthesis/Exocytosis
	PolyHydroxylated/Carbonylated Lipophile (Compact)												
	3-Isobutyl-1-Methylxanthine (IBMX)	C10H14N4O2	0.47	2.951E+00	222	198	0.71	69	0 (Poly C=O)	0.7	CM & CM Receptor Aqueous Pore	CM Perturbomodulation & 1ary Indirect Shift Pressuromodulation	Chromatin DNA Protein Synthesis/Exocytosis
	Circumferentially PolyHydroxylated/Carbonylated/Etheroylated Lipophile [Non-Compact (>Pore Size)]												
	Forskolin	C22H34O7	1.36	2.291E+01	411	396	0.90	113	0 (PolyOH cum C=O)	1.5	CM Receptor Alpha Helix Isophilic Aqueous Pore	Receptor External Hydroxymodulation -> (Pseudo) 3ary Indirect Shift Pressuromodulation	CM Interaction Receptor Endocytosis [Mitogenesis]
	Paclitaxel (Taxol)	C47H51NO14	3.54	3.467E+03	854	764	1.12	221	0 (PolyOH cum C=O + 3 Benzyl Rings)	3.2	CM Receptor Alpha Helix Isophilic Aqueous Pore	Receptor External Hydroxymodulation	CM Interaction Receptor Endocytosis & Microtubular Network Disruption
	Colchicine	C47H51NO17	1.42	2.630E+01	399	364	0.87	83	0 (PolyOCH3 cum Di-C=O)	1.6	CM Receptor Alpha Helix Isophilic Aqueous Pore	Receptor External Hydroxymodulation	CM Interaction Receptor Endocytosis & Microtubular Network Disruption

PI = (CM) Phospholipid Incorporating
