# Supplementary figures and images for: Pressuromodulation at the cell membrane as the basis for small molecule hormone and peptide regulation of cellular and nuclear function
Source: J Transl Med. 2015 Nov 26;13:372. doi: 10.1186/s12967-015-0707-6 (PMC4660824; doi:10.1186/s12967-015-0707-6)

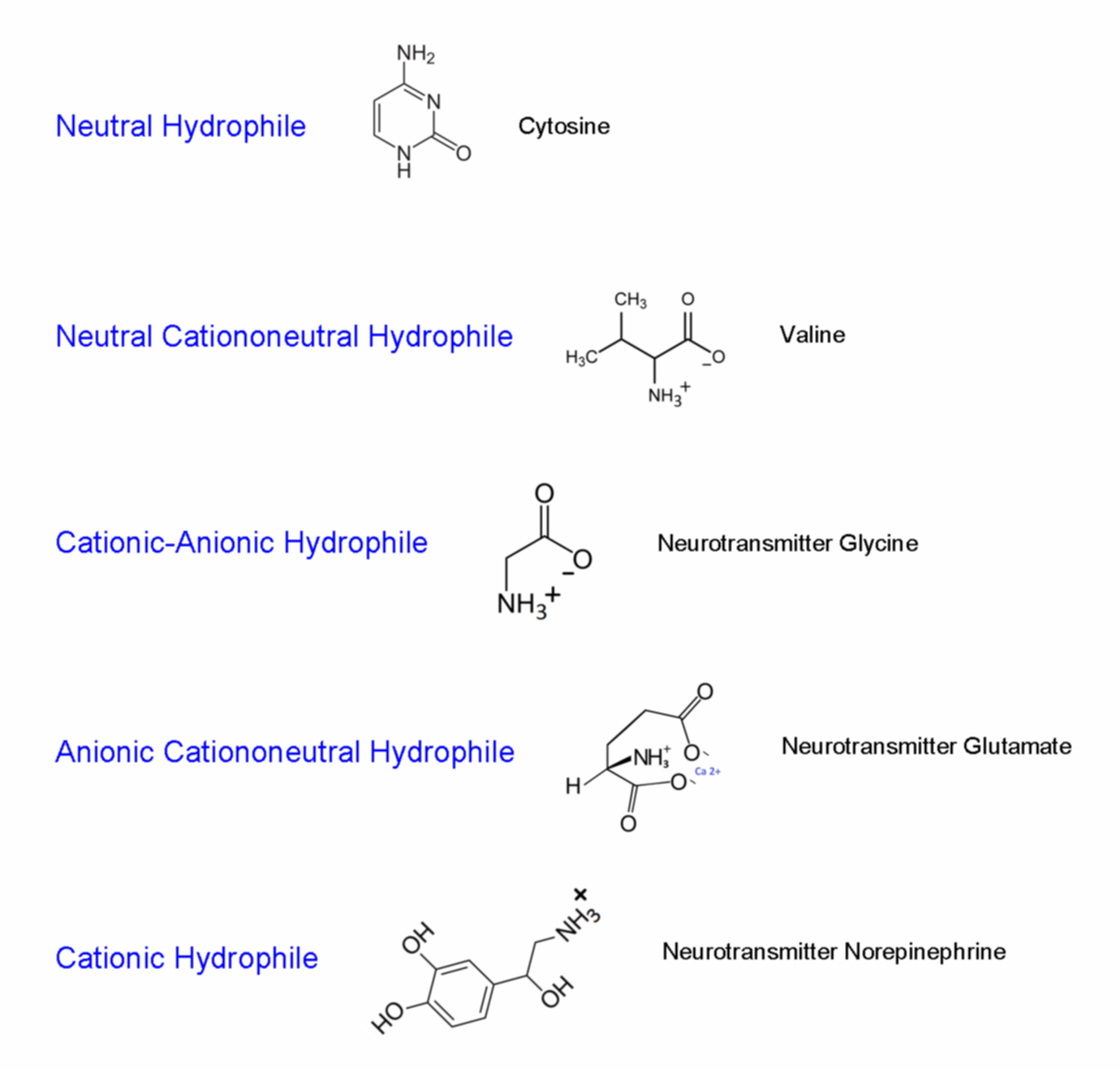

Supplement: Supplementary file 2 — 10.1186/s12967-015-0707-6 Small molecule hydrophiles. [file 12967_2015_707_MOESM2_ESM.jpeg]

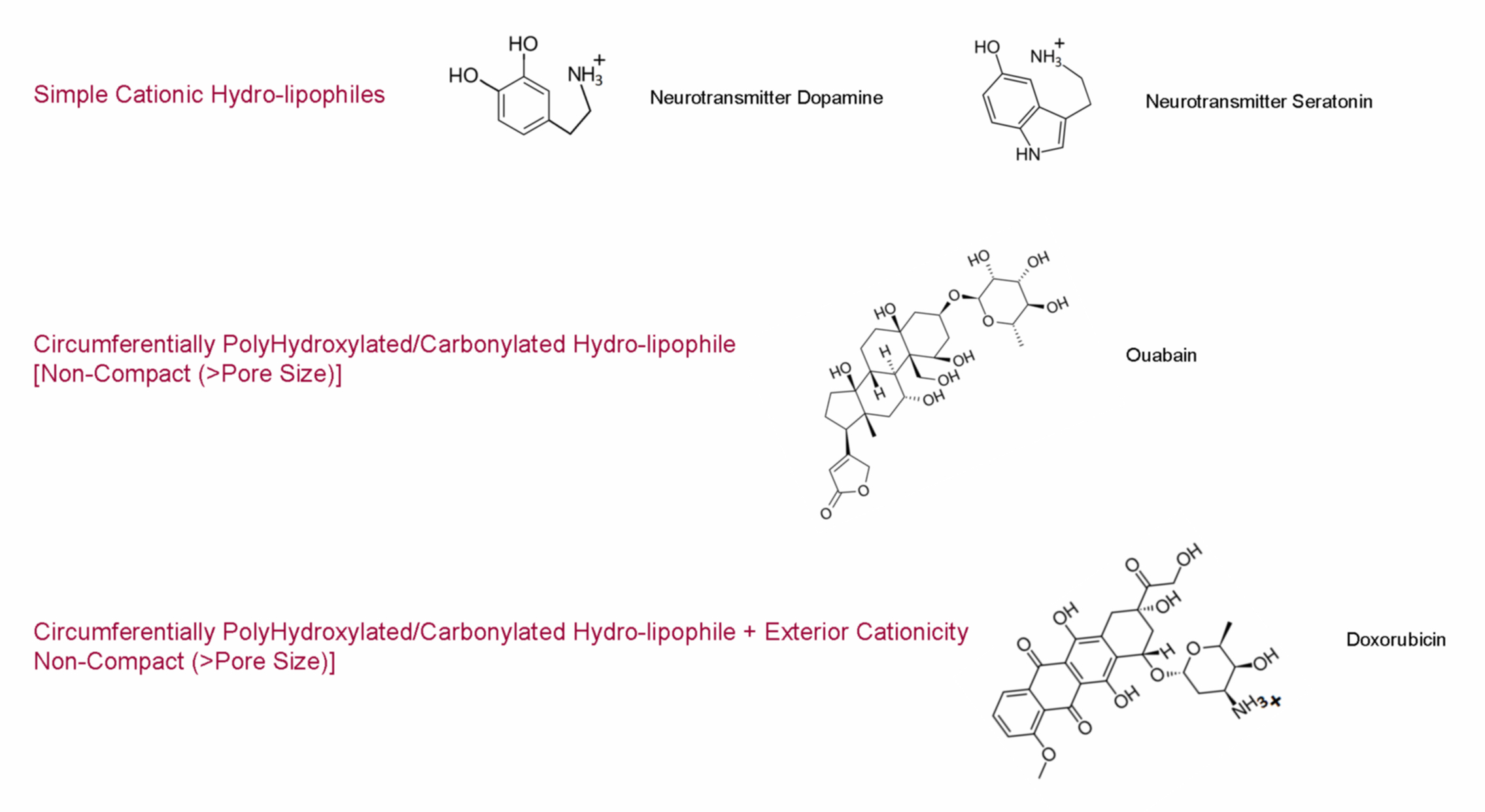

Supplement: Supplementary file 3 — 10.1186/s12967-015-0707-6 Small molecule hydro-lipophiles. [file 12967_2015_707_MOESM3_ESM.jpg]

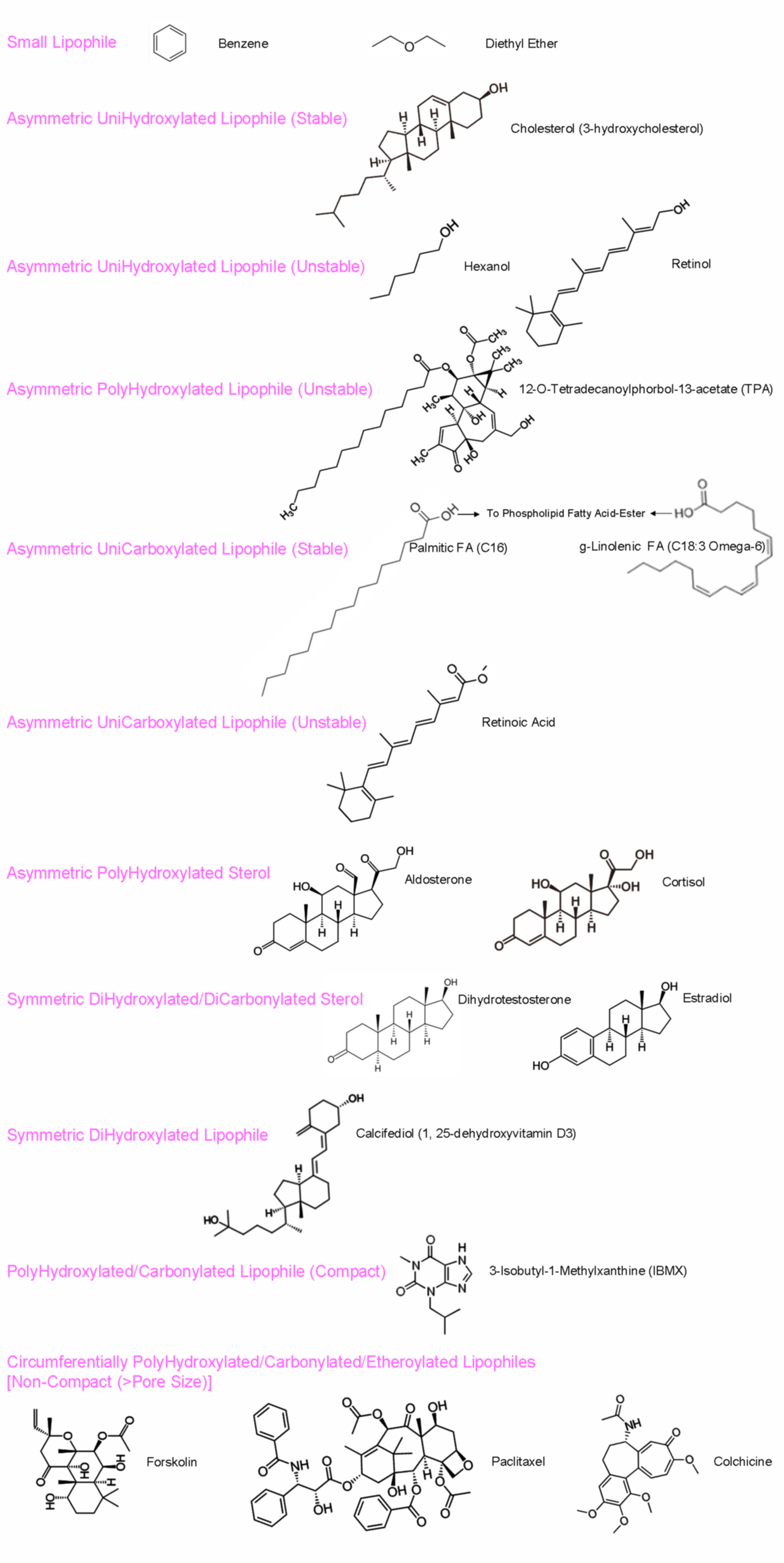

Supplement: Supplementary file 4 — 10.1186/s12967-015-0707-6 Small molecule lipophiles. [file 12967_2015_707_MOESM4_ESM.jpg]
