# Supplementary material for: Pressuromodulation at the cell membrane as the basis for small molecule hormone and peptide regulation of cellular and nuclear function
Source: J Transl Med. 2015 Nov 26;13:372. doi: 10.1186/s12967-015-0707-6 (PMC4660824; doi:10.1186/s12967-015-0707-6)
Supplement: Supplementary file 5 — 10.1186/s12967-015-0707-6 Complete Table 1 in Supplemental File Format. [file 12967_2015_707_MOESM5_ESM.pdf]

Table 1. Conservation of Small Biomolecule, Peptide and Non-Small Molecule Non-Peptide Function

TYPE	SUB-TYPE	EXAMPLE(S)	CELL MEMBRANE (CM) or CM RECEPTOR	EFFECT AT CELL MEMBRANE (CM) RECEPTOR	INTRACELLULAR RESULT OF EFFECT
SMALL MOLECULE					
Small Molecule Hydrophile	Neutral Hydrophile	H_2O, Nitrogenous Bases	CM Receptor Aqueous Pore	n/a (Subcellular Interaction, including Nuclear/Mitochondrial)	CM Receptor/Nuclear/Mitoch Aqueous Pore Permeation -> DNA/RNA
	Neutral Cationoneutral Hydrophile	Apolar Amino Acids (ie Valine, Alanine, Leucine, Isoleucine, etc)	CM Receptor Aqueous Pore	n/a (Subcellular Interaction, including Nuclear/Mitochondrial)	CM Receptor/Nuclear/Mitoch Aqueous Pore Permeation -> Protein
	Cationic-Anionic Hydrophile	Neurotransmitter (Glycine, GABA) (IS 1+ 1-)	CM Receptor Aqueous Pore	Receptor Aqueous Pore Non-Cationomodulation (Isomodulation) & CM Non-Depolarization	CM Receptor Aqueous Pore Permeation
	Anionic Cationoneutral Hydrophile	Neurotransmitter Glutamate (IS 1- 1-)	Peri-CM/Peri-CM Receptor Aqueous Pore	Ca^{2+} Pseudo-Association	Vesicular Non-Auto-Endocytosis
	Cationic Hydrophile	Neurotransmitters Norepinephrine (1+), Acetylcholine (1+)	CM Receptor Aqueous Pore	Receptor Aqueous Pore Internal Cationomodulation & CM Depolarization	Depolarization
	Cationic Hydrophile	Neurotransmitters Norepinephrine (1+), Acetylcholine (1+)	CM	Direct CM Cationiomodulation (Poly IS 1+)	Vesicular Endocytosis
	Cationic Hydrophile	Histamine (IS 1+ 1+) @ Acidic pH	CM	Direct CM Cationiomodulation (2+)	Vesicular Auto-Endocytosis
Small Molecule Hydro-lipophile	Simple Cationic Hydro-lipophile	Neurotransmitters Dopamine (1+), Seratonin (1+) & Histamine (1+)	CM Receptor Protein	Receptor External Cationiomodulation & CM Non-depolarization	Non-depolarization
	Simple Cationic Hydro-lipophile	Neurotransmitters Dopamine (1+), Seratonin (1+) & Histamine (1+)	CM	Direct CM Cationiomodulation (Poly IS 1+)	Vesicular Endocytosis
	Circumferentially PolyHydroxylated/Carbonylated Hydro-lipophile [Non-Compact (>Pore Size)]	ie Ouabain	CM Receptor Alpha Helix Isophilic Aqueous Pore (ie Na/K ATPase)	Receptor External Hydroxymodulation -> (Pseudo) 3ary Indirect Shift Pressuromodulation	CM Interaction Receptor Endocytosis [Mitogenesis]
	Circumferentially PolyHydroxylated/Carbonylated Hydro-lipophile + Exterior Cationicity [Non-Compact (>Pore Size)]	ie Doxorubicin (1+)	CM Receptor Alpha Helix Isophilic Aqueous Pore (ie Na/K ATPase)	Receptor External Hydroxymodulation	CM Interaction Receptor Endocytosis & Cationicity (1+)-Mediated Mitochondrial Toxicity
Small Molecule Lipophile	Small Lipophile	ie Benzene, Diethyl Ether	CM Receptor Aqueous Pore	Mitochondrial Membrane Perturbomodulation -> 1ary Indirect Shift Pressuromodulation	Chromatin DNA Protein Synthesis/Exocytosis
	Asymmetric UniHydroxylated Lipophile (Stable)	Cholesterol (3-hydroxycholesterol), ie Cholecalciferol (3-hydroxyvitamin D3)	CM	CM Incorporopressuromodulation	Baseline Chromatin DNA Protein Synthesis/Exocytosis
	Asymmetric UniHydroxylated Lipophile (Unstable)	ie Hexan-1-ol, Retinol	CM	CM Perturbomodulation -> 1ary Indirect Shift Pressuromodulation	Chromatin DNA Protein Synthesis/Exocytosis
	Asymmetric PolyHydroxylated Lipophile (Unstable)	ie Phorbol Ester 12-O-Tetradecanoylphorbol-13-acetate (TPA)	CM	CM Perturbomodulation -> 1ary Indirect Shift Pressuromodulation	Chromatin DNA Protein Synthesis/Exocytosis
	Asymmetric UniCarboxylated Lipophile (Stable)	Saturated Fatty Acid -> Ester	CM	CM Incorporopressuromodulation	Baseline Chromatin DNA Protein Synthesis/Exocytosis
	Asymmetric UniCarboxylated Lipophile (Stable)	Polyunsaturated Fatty Acid -> Ester (ie Omega-3/6)	CM	CM Incorporonegativopressuromodulation	n/a
	Asymmetric UniCarboxylated Lipophile (Unstable)	Non-Fatty Acid (ie Retinoic Acid)	CM	CM Perturbomodulation -> 1ary Indirect Shift Pressuromodulation	Chromatin DNA Protein Synthesis/Exocytosis
	Asymmetric PolyHydroxylated Sterol	Aldosterone, Dexamethasone, Cortisol	CM Receptor Protein	Receptor Stabilizing Shift Pressuromodulation	Chromatin DNA Protein Synthesis/Exocytosis
	Symmetric Di or TriHydroxylated/DiCarbonylated Sterol	Testosterone, Estrogen, Progesterone	CM Receptor Protein	Receptor Stabilizing Shift Pressuromodulation	Chromatin DNA Protein Synthesis/Exocytosis
	Symmetric DiHydroxylated Lipophile	ie Calcifediol (1,25-dihydroxyvitamin D3)	CM	CM Perturbomodulation -> 1ary Indirect Shift Pressuromodulation	Chromatin DNA Protein Synthesis/Exocytosis
	PolyHydroxylated/Carbonylated Lipophile (Compact)	ie 3-Isobutyl-1-Methylxanthine (IBMX)	CM & CM Receptor Aqueous Pore	CM Perturbomodulation -> 1ary Indirect Shift Pressuromodulation	Chromatin DNA Protein Synthesis/Exocytosis
	Circumferentially PolyHydroxylated/Carbonylated Lipophile [Non-Compact (>Pore Size)]	ie Forskolin	CM Receptor Alpha Helix Isophilic Aqueous Pore	Receptor External Hydroxymodulation -> (Pseudo) 3ary Indirect Shift Pressuromodulation	CM Interaction Receptor Endocytosis [Mitogenesis]
	Circumferentially PolyHydroxylated/Carbonylated/Etheroylated Lipophile [Non-Compact (>Pore Size)]	ie Paclitaxel (Taxol), Colchicine	CM Receptor Alpha Helices	Receptor External Hydroxymodulation	CM Interaction Receptor Endocytosis & Microtubular Network Disruption
NON-SMALL MOLECULE NON-PEPTIDE	polyPhospholipid polySaccharide	Lipopolysaccharide (LPS)	CM	CM Perturbomodulation -> 1ary Indirect Shift Pressuromodulation	Chromatin DNA Protein Synthesis/Exocytosis
PEPTIDE	Small (Non-Alpha Non-Beta Helix) Peptide	Atrial Natriuretic Peptide (ANP) Monomer (1+ IS 1+)	CM Receptor Protein Monomer	Receptor External Cationiomodulation (2+) -> 3ary Indirect Shift Pressuromodulation	Cationic (2+) CM Interaction Receptor Endocytosis [Mitogenesis]
	Small (Non-Alpha Non-Beta Helix) Peptide	Bradykinin Monomer (1-9 AAs) (1+ IS 1+ SS 1+)	CM Receptor Protein Monomer	Receptor External Cationiomodulation (2+) -> 3ary Indirect Shift Pressuromodulation	Cationic (2+) CM Interaction Receptor Endocytosis [Mitogenesis]
	Small (Non-Alpha Non-Beta Helix) Peptide	Des-Arg9 Bradykinin Monomer (1-8 AAs) (1+ IS 1+)	CM Receptor Protein Monomer	Receptor External Cationiomodulation (2+) -> 3ary Indirect Shift Pressuromodulation	Cationic (2+) CM Interaction Receptor Endocytosis [Mitogenesis]
	Small (Non-Alpha Non-Beta Helix) Peptide	Sulfate Neutralized Lys-Bradykinin (Kallidin) Monomer (1-10 AAs) (IS 3+ -> 1+)	CM Receptor Protein Monomer	Receptor Stabilizing Shift Pressuromodulation	Chromatin DNA Protein Synthesis/Exocytosis
	Small (Non-Alpha Non-Beta Helix) Peptide	Vasopressin Arginine (Anti-Diuretic Hormone: ADH) Monomer (1+)	CM Receptor Protein Monomer	Receptor Stabilizing Shift Pressuromodulation	Chromatin DNA Protein Synthesis/Exocytosis
	Small (Non-Alpha Non-Beta Helix) Peptide	Neurotensin Monomer (1+)	CM Receptor Protein Monomer	Receptor Stabilizing Shift Pressuromodulation	Chromatin DNA Protein Synthesis/Exocytosis
	Small (Non-Alpha Non-Beta Helix) Peptide	Angiotensin II Monomer	CM Receptor Protein Monomer	Receptor Stabilizing Shift Pressuromodulation	Chromatin DNA Protein Synthesis/Exocytosis
	Small (Non-Alpha Non-Beta Helix) Peptide	Ps4 Thyrotropin Releasing Hormone (TRH) Monomer	CM Receptor Protein Monomer	Receptor Stabilizing Shift Pressuromodulation	Chromatin DNA Protein Synthesis/Exocytosis
	Small (Non-Alpha Non-Beta Helix) Peptide	Somatostatin (Growth Hormone Release Inhibiting Peptide: GHRIP) Monomer	CM Receptor Protein Monomer	Receptor Stabilizing Shift Pressuromodulation	Chromatin DNA Protein Synthesis/Exocytosis
	Small (Non-Alpha Non-Beta Helix) Peptide	Oxytocin Monomer	CM Receptor Protein Monomer	Receptor Stabilizing Shift Pressuromodulation	Chromatin DNA Protein Synthesis/Exocytosis
	Short MonoAlpha Helix	Glucagon Monomer	CM Receptor Protein Monomer	Receptor Stabilizing Shift Pressuromodulation	Chromatin DNA Protein Synthesis/Exocytosis
	Short MonoAlpha Helix-Loop-Short 2-Way Beta Helix	Adrenocorticotropic Hormone (ACTH) Monomer	CM Receptor Protein Monomer -> Dimer	Receptor Stabilizing Shift Pressuromodulation	Chromatin DNA Protein Synthesis/Exocytosis
	Long MonoAlpha Helix	Parathyroid Hormone (PTH)/PTH Releasing Peptide (PTHrP) Monomer	CM Receptor Protein Monomer	Receptor Stabilizing Shift Pressuromodulation	Chromatin DNA Protein Synthesis/Exocytosis
	Long MonoAlpha Helix-Loop-Short 2-Way Beta Helix	Pro-ACTH Pro-opiomelanocortin (POMC) Monomer	CM Receptor Protein Monomer -> Dimer	Receptor Stabilizing Shift Pressuromodulation	Chromatin DNA Protein Synthesis/Exocytosis
	Short MonoAlpha Helix-Loop-Short MonoAlpha Helix	Adrenocorticotrophin Releasing Hormone/Factor (CRH/F) Monomer	CM Receptor Protein Dimer (2 su: 3 su)	Dual Receptor Stabilizing Shift Pressuromodulation	Chromatin DNA Protein Synthesis/Exocytosis
	Short MonoAlpha Helix-Loop-Short MonoAlpha Helix	Insulin Monomer	CM Receptor Protein Dimer (2 su: 3 su)	Dual Receptor Stabilizing Shift Pressuromodulation	Chromatin DNA Protein Synthesis/Exocytosis
	Short MonoAlpha Helix-Loop-Short MonoAlpha Helix-Loop-Short MonoAlpha Helix	Osteocalcin Monomer	CM Receptor Protein Dimer (2 su: 3 su)	Dual Receptor Stabilizing Shift Pressuromodulation	Chromatin DNA Protein Synthesis/Exocytosis
	Short MonoAlpha Helix-Loop-Short MonoAlpha Helix-Loop-Short MonoAlpha Helix	Insulin-like Growth Factor-1 (IGF1/II; Somatomedin C) Monomer	CM Receptor Protein Dimer (2 su: 3 su)	Dual Receptor Stabilizing Shift Pressuromodulation	Chromatin DNA Protein Synthesis/Exocytosis
	Short MonoAlpha Helix-Loop-Short MonoAlpha Helix-Loop-Short MonoAlpha Helix	Prolactin (PRL) Releasing Hormone/Factor (PRLRH/Factor) Monomer	CM Receptor Protein Dimer (2 su: 3 su)	Dual Receptor Stabilizing Shift Pressuromodulation	Chromatin DNA Protein Synthesis/Exocytosis
	Short MonoAlpha Helix-Loop-Short MonoAlpha Helix-Loop-Short MonoAlpha Helix	Growth Hormone Releasing Hormone/Factor (GHRH/F) Monomer	CM Receptor Protein Dimer (2 su: 3 su)	Dual Receptor Stabilizing Shift Pressuromodulation	Chromatin DNA Protein Synthesis/Exocytosis
	Short MonoAlpha Helix-Loop-Short MonoAlpha Helix-Loop-Short MonoAlpha Helix	Gonadotropin Releasing Hormone/Factor (GnRH/F) Monomer	CM Receptor Protein Dimer (2 su: 3 su)	Dual Receptor Stabilizing Shift Pressuromodulation	Chromatin DNA Protein Synthesis/Exocytosis
	Aligned MultiAlpha Helix	Interleukin-3 (IL-3) Monomer	CM Receptor Protein Dimer (2 su: 2 su)	Dual Receptor Stabilizing Shift Pressuromodulation	Chromatin DNA Protein Synthesis/Exocytosis
	Aligned MultiAlpha Helix	Interleukin-7 (IL-7) Monomer	CM Receptor Protein Dimer (2 su: 2 su)	Dual Receptor Stabilizing Shift Pressuromodulation	Chromatin DNA Protein Synthesis/Exocytosis
	Aligned MultiAlpha Helix	Interleukin-12 (IL-12) Monomer	CM Receptor Protein Dimer (2 su: 2 su)	Dual Receptor Stabilizing Shift Pressuromodulation	Chromatin DNA Protein Synthesis/Exocytosis
	Aligned MultiAlpha Helix	Interleukin-23 (IL-23) Monomer	CM Receptor Protein Dimer (2 su: 2 su)	Dual Receptor Stabilizing Shift Pressuromodulation	Chromatin DNA Protein Synthesis/Exocytosis
	Aligned MultiAlpha Helix	Prolactin (PRL) Monomer	CM Receptor Protein Dimer (2 su: 2 su)	Dual Receptor Stabilizing Shift Pressuromodulation	Chromatin DNA Protein Synthesis/Exocytosis
	Aligned MultiAlpha Helix	Growth Hormone (GH) Monomer	CM Receptor Protein Dimer (2 su: 2 su)	Dual Receptor Stabilizing Shift Pressuromodulation	Chromatin DNA Protein Synthesis/Exocytosis
	Aligned MultiAlpha Helix	Erythropoietin (Ep) Monomer	CM Receptor Protein Dimer (2 su: 2 su)	Dual Receptor Stabilizing Shift Pressuromodulation	Chromatin DNA Protein Synthesis/Exocytosis
	Aligned MultiAlpha Helix	Interferon gamma (INF-g) Homodimer	CM Receptor Protein Trimer (3 su: 3 su: <3 su>)	Dual-to-Tri Receptor Stabilizing Shift Pressuromodulation	Chromatin DNA Protein Synthesis/Exocytosis
	Semi-Aligned MultiAlpha Helix cum Short MonoAlpha Helix	Interleukin-2 (IL-2) Monomer	CM Receptor Protein Dimer (3 su: 2 su)	Dual Receptor Stabilizing Shift Pressuromodulation	Chromatin DNA Protein Synthesis/Exocytosis
	Semi-Aligned MultiAlpha Helix cum Short MonoAlpha Helix	Interleukin-6 (IL-6) Monomer	CM Receptor Protein Dimer (3 su: 2 su)	Dual Receptor Stabilizing Shift Pressuromodulation	Chromatin DNA Protein Synthesis/Exocytosis
	Semi-Aligned MultiAlpha Helix cum Short MonoBeta 2-Way Helix	Interleukin-4 (IL-4) Monomer	CM Receptor Protein Dimer (4 su: 2 su)	Dual Receptor Stabilizing Shift Pressuromodulation	Chromatin DNA Protein Synthesis/Exocytosis
	Semi-Aligned MultiAlpha Helix cum Short MonoBeta 2-Way Helix	Interleukin-13 (IL-13) Monomer	CM Receptor Protein Dimer (4 su: 2 su)	Dual Receptor Stabilizing Shift Pressuromodulation	Chromatin DNA Protein Synthesis/Exocytosis
	Semi-Aligned MultiAlpha Helix cum Short MonoBeta 2-Way Helix	Interleukin-5 (IL-5) Monomer	CM Receptor Protein Dimer (4 su: 2 su)	Dual Receptor Stabilizing Shift Pressuromodulation	Chromatin DNA Protein Synthesis/Exocytosis
	Semi-Aligned MultiAlpha Helix cum Short MonoBeta 2-Way Helix	Interleukin-15 (IL-15) Monomer	CM Receptor Protein Dimer (4 su: 2 su)	Dual Receptor Stabilizing Shift Pressuromodulation	Chromatin DNA Protein Synthesis/Exocytosis
	Semi-Aligned MultiAlpha Helix cum Short MonoBeta 2-Way Helix	Interleukin-20 (IL-20) Monomer	CM Receptor Protein Dimer (4 su: 2 su)	Dual Receptor Stabilizing Shift Pressuromodulation	Chromatin DNA Protein Synthesis/Exocytosis
	Semi-Aligned MultiAlpha Helix cum Short MonoBeta 2-Way Helix	Granulocyte Monocyte-Colony Stimulating Factor (GM-CSF) Monomer	CM Receptor Protein Dimer (4 su: 2 su)	Dual Receptor Stabilizing Shift Pressuromodulation	Chromatin DNA Protein Synthesis/Exocytosis
	Semi-Aligned MultiAlpha Helix cum Short MonoAlpha Helix cum Short Loop	Leukemia Inhibitory Factor (LIF)/Oncostatin (OSM) Monomer	CM Receptor Protein Dimer (5 su: 3 su)	Dual Receptor Stabilizing Shift Pressuromodulation	Chromatin DNA Protein Synthesis/Exocytosis
	Semi-Aligned MultiAlpha Helix-Loop-Short MonoAlpha Helix-Loop-Short MonoBeta 2-Way Helix	Granulocyte-Colony Stimulating Factor (G-CSF)/Macrophage (Mouse)-Colony Stimulating Factor (M-CSF) Homodimer	CM Receptor Protein Dimer (5 su: 3 su)	Dual Receptor Stabilizing Shift Pressuromodulation	Chromatin DNA Protein Synthesis/Exocytosis
	Short MonoAlpha Helix (Neutral) cum Compact Loose Non-Aligned 3-Way Beta-X-2-Way Beta Helix (Cationic)	Sulfate Neutralized Interleukin-16 (IL-16) Monomer (IS 3+ -> 1+)	CM Receptor Protein (CD4) Monomer	External Cationiomodulation & Receptor Stabilizing Shift Pressuromodulation	Extracellular Matrix Interaction & Chromatin DNA Protein Synthesis/Exocytosis
	Short MonoAlpha Helix (Neutral) cum Compact Loose Aligned 3-Way Beta Helix (Cationic)	Sulfate Neutralized Macrophage Inflammatory Protein-1 beta (MIP-1 beta: CCL20) Monomer (IS 3+ -> 1+)	CM Receptor Protein (CCR5) Monomer	External Cationiomodulation & Receptor Stabilizing Shift Pressuromodulation	Extracellular Matrix Interaction & Chromatin DNA Protein Synthesis/Exocytosis
	Short MonoAlpha Helix (Neutral) cum Compact Loose Aligned 3-Way Beta Helix (Cationic)	Sulfate Neutralized CCL3/CCL4/CCL5 (RANTES) Monomer (IS 3+ -> 1+)	CM Receptor Protein (CCR5) Monomer	External Cationiomodulation & Receptor Stabilizing Shift Pressuromodulation	Extracellular Matrix Interaction & Chromatin DNA Protein Synthesis/Exocytosis
	Short Angled MonoAlpha Helix (Neutral) cum Compact Loose Aligned 3-Way Beta Helix (Cationic)	Sulfate Neutralized SDF-1 (CXCL-12) Monomer (IS 3+ -> 1+)	CM Receptor Protein (CXCR4) Monomer	External Cationiomodulation & Receptor Stabilizing Shift Pressuromodulation	Extracellular Matrix Interaction & Chromatin DNA Protein Synthesis/Exocytosis
	Short Angled MonoAlpha Helix (Neutral) cum Compact Loose Aligned 3.5-Way Beta Helix (Cationic)	Sulfate Neutralized Interleukin-8 (IL-8) Monomer (IS 3+ -> 1+)	CM Receptor Protein (CXCR1) Monomer	External Cationiomodulation & Receptor Stabilizing Shift Pressuromodulation	Extracellular Matrix Interaction & Chromatin DNA Protein Synthesis/Exocytosis
	Compact Loose Non-Aligned MultiBeta Helix (Cationic) [+/- Alpha Helix Knob (Neutral)]	Sulfate Neutralized Fibroblast Growth Factor (FGF-19/FGF-2) Monomer x2 (IS 3+ -> 1+: IS 3+ -> 1+)	CM Receptor Protein Monomer (3 su) -> Dimer (3 su: 3 su)	External Cationiomodulation & Dual Receptor Stabilizing Shift Pressuromodulation	Extracellular Matrix Interaction & Chromatin DNA Protein Synthesis/Exocytosis
	Compact Loose Non-Aligned MultiBeta Helix (Cationic) [+/- Alpha Helix Knob (Neutral)]	Sulfate Neutralized Hepatocyte Growth Factor alpha (HGF alpha; Scatter Factor) Monomer x2 (IS 3+ -> 1+: IS 3+ -> 1+)	CM Receptor Protein Monomer (3 su) -> Dimer (3 su: 3 su)	External Cationiomodulation & Dual Receptor Stabilizing Shift Pressuromodulation	Extracellular Matrix Interaction & Chromatin DNA Protein Synthesis/Exocytosis
	Compact Loose Non-Aligned MultiBeta Helix (Cationic) [+/- Alpha Helix Knob (Neutral)]	Sulfate Neutralized Epidermal Growth Factor (EGF) Monomer x2 (IS 3+ -> 1+: IS 3+ -> 1+)	CM Receptor Protein Monomer (3 su) -> Dimer (3 su: 3 su)	External Cationiomodulation & Dual Receptor Stabilizing Shift Pressuromodulation	Extracellular Matrix Interaction & Chromatin DNA Protein Synthesis/Exocytosis
	Compact Loose Non-Aligned MultiBeta Helix (Cationic) [+/- Alpha Helix Knob (Neutral)]	Sulfate Neutralized Interleukin-1 alpha (IL-1 alpha) Monomer x2 (IS 3+ -> 1+: IS 3+ -> 1+)	CM Receptor Protein Monomer (3 su) -> Dimer (3 su: 3 su)	External Cationiomodulation & Dual Receptor Stabilizing Shift Pressuromodulation	Extracellular Matrix Interaction & Chromatin DNA Protein Synthesis/Exocytosis
	Compact Loose Non-Aligned MultiBeta Helix (Cationic) [+/- Alpha Helix Knob (Neutral)]	Sulfate Neutralized Interleukin-1 beta (IL-1 beta) Monomer x2 (IS 3+ -> 1+: IS 3+ -> 1+)	CM Receptor Protein Monomer (3 su) -> Dimer (3 su: 3 su)	External Cationiomodulation & Dual Receptor Stabilizing Shift Pressuromodulation	Extracellular Matrix Interaction & Chromatin DNA Protein Synthesis/Exocytosis
	Compact Tight Semi-Aligned MultiBeta Helix (Cationic)	Tumor Necrosis Factor alpha (TNF alpha) Homotrimer (SS 1+)	CM Receptor Protein Trimer	Tri Receptor Internal Pseudo-Cationomodulation -> 2ary Indirect Shift Pressuromodulation	Chromatin DNA Protein Synthesis/Exocytosis [Mitogenesis]
	Compact Tight Aligned MultiBeta Helix (Cationic)	Adiponectin Homotrimer (SS 1+)	CM Receptor Protein Trimer	Tri Receptor Internal Pseudo-Cationomodulation -> 2ary Indirect Shift Pressuromodulation	Chromatin DNA Protein Synthesis/Exocytosis [Mitogenesis]
	Compact Tight Aligned MultiBeta Helix (Cationic)	RANKL Homotrimer (SS 1+)	CM Receptor Protein Trimer	Tri Receptor Internal Pseudo-Cationomodulation -> 2ary Indirect Shift Pressuromodulation	Chromatin DNA Protein Synthesis/Exocytosis [Mitogenesis]
	Aligned Long MultiBeta Helix (Cationic)	Sulfate Neutralized Thyroid Stimulating Hormone alpha & beta (TSH alpha & beta) Heterodimer (IS 3+ -> 1+: IS 3+ -> 1+)	CM Receptor Protein Trimer	Tri Receptor Stabilizing Shift Pressuromodulation & External Cationiomodulation	Chromatin DNA Protein Synthesis/Exocytosis & Extracellular Matrix Interaction
	Aligned Long MultiBeta Helix (Cationic)	Sulfate Neutralized Luteinizing Hormone alpha & beta (LH alpha & beta) Heterodimer (IS 3+ -> 1+: IS 3+ -> 1+)	CM Receptor Protein Trimer	Tri Receptor Stabilizing Shift Pressuromodulation & External Cationiomodulation	Chromatin DNA Protein Synthesis/Exocytosis & Extracellular Matrix Interaction
	Aligned Long MultiBeta Helix (Cationic)	Sulfate Neutralized Follicle Stimulating Hormone alpha & beta (FSH alpha & beta) Heterodimer (IS 3+ -> 1+: IS 3+ -> 1+)	CM Receptor Protein Trimer	Tri Receptor Stabilizing Shift Pressuromodulation & External Cationiomodulation	Chromatin DNA Protein Synthesis/Exocytosis & Extracellular Matrix Interaction
	Aligned Long MultiBeta Helix (Cationic)	Sulfate Neutralized Human Chorionic Gonadotropin alpha & beta (HCG alpha & beta) Heterodimer (IS 3+ -> 1+: IS 3+ -> 1+)	CM Receptor Protein Trimer	Tri Receptor Stabilizing Shift Pressuromodulation & External Cationiomodulation	Chromatin DNA Protein Synthesis/Exocytosis & Extracellular Matrix Interaction
	Aligned Long MultiBeta Helix (Cationic)	Sulfate Neutralized Brain-Derived Neurotrophic Factor (BDNF) Homodimer (IS 3+ -> 1+: IS 3+ -> 1+)	CM Receptor Protein Trimer	Tri Receptor Stabilizing Shift Pressuromodulation & External Cationiomodulation	Chromatin DNA Protein Synthesis/Exocytosis & Extracellular Matrix Interaction
	Aligned Long MultiBeta Helix (Cationic)	Sulfate Neutralized Nerve Growth Factor beta (NGFb) Homodimer (IS 3+ -> 1+: IS 3+ -> 1+)	CM Receptor Protein Trimer	Tri Receptor Stabilizing Shift Pressuromodulation & External Cationiomodulation	Chromatin DNA Protein Synthesis/Exocytosis & Extracellular Matrix Interaction
	Aligned Long MultiBeta Helix (Cationic)	Sulfate Neutralized Neurotrophins (NTs) Homodimer (IS 3+ -> 1+: IS 3+ -> 1+)	CM Receptor Protein Trimer	Tri Receptor Stabilizing Shift Pressuromodulation & External Cationiomodulation	Chromatin DNA Protein Synthesis/Exocytosis & Extracellular Matrix Interaction
	Aligned Long MultiBeta Helix (Cationic) cum Short MonoAlpha Helix (Neutral)	Transforming Growth Factor beta (TGF beta) Homodimer (SS 1+)	CM Receptor Protein Quatramer	Quad Receptor Internal Pseudo-Cationomodulation -> 2ary Indirect Shift Pressuromodulation	Chromatin DNA Protein Synthesis/Exocytosis [Mitogenesis]
	Aligned Long MultiBeta Helix (Cationic) cum Short MonoAlpha Helix (Neutral)	Bone Morphogenic Protein-2/7 (BMP-2/7) Homodimer (SS 1+)	CM Receptor Protein Quatramer	Quad Receptor Internal Pseudo-Cationomodulation -> 2ary Indirect Shift Pressuromodulation	Chromatin DNA Protein Synthesis/Exocytosis [Mitogenesis]
	Aligned Long MultiBeta Helix (Cationic) cum Short MonoAlpha Helix (Neutral)	Platelet Derived Growth Factor-BB (PDGF-BB) Homodimer (SS 1+)	CM Receptor Protein Quatramer	Quad Receptor Internal Pseudo-Cationomodulation -> 2ary Indirect Shift Pressuromodulation	Chromatin DNA Protein Synthesis/Exocytosis [Mitogenesis]
	Aligned Long MultiBeta Helix (Cationic) cum Short Angled MonoAlpha Helix (Neutral)	Placenta Growth Factor (PLGF) Homodimer (SS 1+)	CM Receptor Protein Quatramer	Quad Receptor Internal Pseudo-Cationomodulation -> 2ary Indirect Shift Pressuromodulation	Chromatin DNA Protein Synthesis/Exocytosis [Mitogenesis]
	Aligned Long MultiBeta Helix (Cationic) cum Short Angled MonoAlpha Helix (Neutral)	Vascular Endothelial Growth Factor-A (VEGF-A)/Vascular Permeability Factor (VPF) Homodimer (1+ IS 1+ SS 1+ IS 1+)	CM Receptor Protein Quatramer	Quad Receptor External Cationiomodulation (2+) -> 3ary Indirect Shift Pressuromodulation	Cationic (2+) CM Interaction Receptor Endocytosis -> Mitogenesis
	Compact Loose Non-Aligned MultiBeta Helix-Intertwined-Non-Aligned MultiAlpha Helix	Diferric Transferrin-(Fe^{3+} -> Fe^{2+})2 Monomers x2 (3+ SS 3+: 3+ SS 3+ -> 2+ SS 2+: 2+ SS 2+)	CM Receptor Protein (TfR) Dimer	Fe^{2+} Receptor External Cationiomodulation (2+) -> 3ary Indirect Shift Pressuromodulation	Cationic Diferric Iron (Fe^{2+}) CM Interaction Receptor Endocytosis -> Mitogenesis
	Long DualAlpha Helix (Neutral) cum Compact Loose Aligned Beta Helix cum MultiShort 2-Way Beta Helix (Cationic)	Hemochromatosis Protein (HPE) Monomers x2 [(1+ IS 1+)n SS (1+ IS 1+)n]	CM Receptor Protein (TfR) Dimer	Transient RSP; External Beta Helix CM Receptor Cationiomodulation (2+) -> 3ary Indirect Shift Pressuromodulation	Cationic (2+) CM Interaction Receptor Endocytosis -> Mitogenesis
	Short MonoAlpha Helix-Loop-Short MonoAlpha Helix-Loop-Short MonoAlpha Helix (Neutral) cum Compact Loose Non-Aligned 5-Way Beta-X-3-Way Beta Helix (Cationic)	Partially Sulfate Neutralized Procollagen I Peptide III C Monomer (IS 4+ -> 2+)	CM Receptor Protein Dimer (3 su: 3 su)	Dual Receptor External Cationiomodulation (2+) -> 3ary Indirect Shift Pressuromodulation	Cationic (2+) CM Interaction Receptor Endocytosis -> Mitogenesis

SS = Sufficient separation of 2+ cationicity in molecular space, which is important, as it precludes Heparan Sulfate neutralization of cationicity, that which requires the presence of >2+ charge insufficiently separated in molecular space

IS = Insufficient separation of 1+ cationicity in molecular space, which is important, as it precludes endocytosis, that which requires the presence of 2+ charge insufficiently separated in molecular space
